# Syphilis and the host: multi-omic analysis of host cellular responses to *Treponema pallidum* provides novel insight into syphilis pathogenesis

**DOI:** 10.3389/fmicb.2023.1254342

**Published:** 2023-09-19

**Authors:** Sean Waugh, Akash Ranasinghe, Alloysius Gomez, Simon Houston, Karen V. Lithgow, Azad Eshghi, Jenna Fleetwood, Kate M. E. Conway, Lisa A. Reynolds, Caroline E. Cameron

**Affiliations:** ^1^Department of Biochemistry and Microbiology, University of Victoria, Victoria, BC, Canada; ^2^University of Victoria-Genome BC Proteomics Centre, Victoria, BC, Canada; ^3^Division of Allergy and Infectious Diseases, Department of Medicine, University of Washington, Seattle, WA, United States

**Keywords:** syphilis, *Treponema pallidum*, proteomics, dissemination, disease symptoms

## Abstract

**Introduction:**

Syphilis is a chronic, multi-stage infection caused by the extracellular bacterium *Treponema pallidum* ssp. *pallidum*. *Treponema pallidum* widely disseminates through the vasculature, crosses endothelial, blood–brain and placental barriers, and establishes systemic infection. Although the capacity of *T. pallidum* to traverse the endothelium is well-described, the response of endothelial cells to *T. pallidum* exposure, and the contribution of this response to treponemal traversal, is poorly understood.

**Methods:**

To address this knowledge gap, we used quantitative proteomics and cytokine profiling to characterize endothelial responses to *T. pallidum*.

**Results:**

Proteomic analyses detected altered host pathways controlling extracellular matrix organization, necroptosis and cell death, and innate immune signaling. Cytokine analyses of endothelial cells exposed to *T. pallidum* revealed increased secretion of interleukin (IL)-6, IL-8, and vascular endothelial growth factor (VEGF), and decreased secretion of monocyte chemoattractant protein-1 (MCP-1).

**Discussion:**

This study provides insight into the molecular basis of syphilis disease symptoms and the enhanced susceptibility of individuals infected with syphilis to HIV co-infection. These investigations also enhance understanding of the host response to *T. pallidum* exposure and the pathogenic strategies used by *T. pallidum* to disseminate and persist within the host. Furthermore, our findings highlight the critical need for inclusion of appropriate controls when conducting *T. pallidum*-host cell interactions using *in vitro-* and *in vivo-*grown *T. pallidum*.

## 1. Introduction

*Treponema pallidum* ssp. *pallidum* (*T. pallidum*), the causative agent of syphilis, is a highly invasive bloodborne bacterial pathogen that causes a chronic and multi-stage infection with an estimated global burden of 19.9 million cases (Rowley et al., 2019). *Treponema pallidum* readily traverses the endothelium, as well as more restrictive barriers such as the blood–brain barrier (BBB) and the placental barrier, to establish a chronic and systemic infection which is lifelong in the absence of antibiotic treatment (LaFond and Lukehart, 2006; Salazar et al., 2007). The clinical presentations of syphilis are diverse, including a characteristic ulcerative chancre at the site of infection during primary syphilis, a disseminated rash during secondary syphilis, and necrotic tissue destruction (gumma) in the tertiary stage of disease (LaFond and Lukehart, 2006; Carlson et al., 2011). With global syphilis cases at a 20 year high in low-, middle-, and high-income countries (Rowley et al., 2019; Spiteri et al., 2019), it is becoming increasingly clear that effective disease control will require augmentation of public health control programs with biomedical prevention strategies. Development of such strategies, which include syphilis vaccine development, requires a comprehensive knowledge of both the pathogenic mechanisms employed by *T. pallidum* to infect the host and the corresponding host cellular response to *T. pallidum* infection, knowledge which is currently limited in the *T. pallidum* field. Previous targeted investigations identified that *T. pallidum* activates endothelial intercellular adhesion molecule 1 (ICAM-1) and fibrin strand formation in human umbilical vein endothelial cells (HUVECs) (Riley et al., 1992), and that *T. pallidum* alters HUVEC vascular endothelial (VE)-cadherin architecture (Lithgow et al., 2021). Herein, we enhance our understanding in this area by performing untargeted multi-omic analyses on host cells exposed to *T. pallidum* to characterize global endothelial responses to this important pathogen.

The vascular endothelium is a vital and dynamic barrier that separates the circulatory system from organs and tissues and is among the first sites of contact for pathogens seeking to establish infection. Consequently, the response of endothelial cells to pathogen contact can be pivotal in the initiation of infection by the pathogen and the initiation of immune responses by the host (Lemichez et al., 2010). Endothelial cells actively contribute to immunity by secreting cytokines to induce signaling pathways that result in the up- and/or down-regulation of inflammation, and by expressing cell adhesion receptors to recruit and enable leukocyte extravasation to organs and tissues during infection. Endothelial cells also secrete extracellular matrix (ECM) components that enable adherence and crosstalk between immune cells, pathogens, and the endothelium (Tomlin and Piccinini, 2018). Specialized endothelial cells, such as those that make up the blood–brain and placental barriers, have additional competencies, including increased expression of tight junction proteins to further protect organs and tissues from infection (Lemichez et al., 2010; Al-Obaidi and Desa, 2018). Endothelial cells must balance barrier function with selective permeability to enable leukocyte movement in and out of the bloodstream under infection conditions. Accordingly, endothelial cells have finely controlled mechanisms that allow leukocytes to traverse both between neighboring endothelial cells (paracellular transport) and directly through endothelial cells (transcellular transport) (Muller, 2013). At sites of inflammation, endothelial cells allow immune cell infiltration by inducing signaling pathways which rearrange the cytoskeleton and loosen endothelial junctions. These endogenous endothelial extravasation mechanisms can be exploited by pathogenic bacteria to allow for deeper access to tissue sites (Galan and Zhou, 2000; Vadeboncoeur et al., 2003; Coureuil et al., 2010; Lemichez et al., 2010; Liu et al., 2018; Káňová et al., 2019).

The current study aims to enhance understanding of the functional consequences of *T. pallidum*-endothelial cell engagement. This was investigated via analyses of the cytokine secretion profile and molecular response of *T. pallidum*-exposed human cerebral microvascular endothelial cells, using the methodologies of cytometric bead arrays (CBAs) and quantitative Stable Isotope Labeling of Amino acids in Cell culture (SILAC) proteomics, respectively. For these investigations, assays were conducted with hCMEC/d3 human cerebral microvascular endothelial cells, a well-characterized cell line that serves as a model of the human BBB (Weksler et al., 2013). Of note, our studies were designed to identify cellular responses raised against *T. pallidum* itself, and to exclude cellular responses raised against host cell contaminants originating from *in vivo* or *in vitro* cultivation of *T. pallidum*. Using this carefully controlled system, our secretomic studies show that exposure of the endothelium to viable *T. pallidum* leads to increased secretion of the pro-inflammatory cytokines interleukin (IL)-6, IL-8, and vascular endothelial growth factor (VEGF), and decreased secretion of monocyte chemoattractant protein-1 (MCP-1). Additionally, our proteomics investigations reveal that endothelial cells exposed to *T. pallidum* have significantly altered expression of ECM components and cytoskeletal regulatory proteins, downregulated expression of proteins related to immune signaling, and differential expression of proteins involved in the necroptotic cell death pathway. Overall, these analyses provide novel insight into the cellular and functional consequences of *T. pallidum*-endothelial cell engagement and identify host cell pathways and processes central to *T. pallidum* pathogenesis.

## 2. Methods

### 2.1. *Treponema pallidum* growth

Outbred male specific pathogen-free (SPF) New Zealand White rabbits (3.0–3.5 kg, Charles River Laboratories, Ontario, Canada) with nonreactive syphilis serological tests (VDRL and FTA-ABS) were used for *in vivo* propagation of *T. pallidum* subsp. *pallidum* Nichols strain as previously described (Lukehart and Marra, 2007). All rabbits were fed antibiotic-free food and water, and were housed at 18–20°C. Animal studies were approved by the local institutional review board under protocol 2016–033 and were conducted in strict accordance with standard accepted principles as set forth by the Canadian Council on Animal Care (CCAC), National Institutes of Health, and the United States Department of Agriculture in facilities accredited by the American Association for the Accreditation of Laboratory Animal Care and the CCAC. Institutional biosafety approval was obtained under biosafety certificate 13170-010. *Treponema pallidum* was cultured *in vitro* with Sf1Ep cells as previously described (Edmondson et al., 2018), with the modification that treponemes were disassociated from Sf1Ep cells using trypsin-free 0.68 mM EDTA for 30 min at 34°C in 1.5% O_2_, 5% CO_2_, 93.5% N_2_ to maintain the integrity of *T. pallidum* outer membrane proteins (Edmondson and Norris, 2021). Bacteria were quantitated by darkfield microscopy (Nikon Eclipse E600; Nikon Canada, Mississauga, Ontario) using a Petroff-Hauser counting chamber (Hauser Scientific, Horsham, PA).

### 2.2. Viable *Treponema pallidum* and infection extract control sample preparations

For the proteomics and CBA experiments, *T. pallidum* was extracted in 10% normal rabbit serum (NRS) in 0.9% NaCl pH 7.0 or *Treponema pallidum* culture medium 2 (TPCM2) formulated as previously described (Edmondson et al., 2018), respectively. Extracts were centrifuged twice at RT at 200 × *g* for 5 min each, followed by 7 min at 400 × *g*, with retention of the *T. pallidum*-containing supernatant each time, after which the supernatant was split into two equal aliquots and centrifuged again at 400 × *g* for 7 min. For the proteomics experiments, the viable treponeme suspension contained in one supernatant (designated viable *T. pallidum* [VTP]) was processed for proteomics analyses as described below. The second supernatant was further processed to remove viable *T. pallidum* and create an optimal comparator control (designated infection extract control [IEC]) that contained a level of host protein contamination that mirrored that found in the VTP. Briefly, this second supernatant aliquot was syringe filtered through a 0.22 μm cellulose acetate filter (Avantor, Allentown, PA) to remove *T. pallidum*, after which the filtrate was added back to the rabbit cellular debris pellet from the preceding centrifugation step that had been washed twice with 10% NRS 0.9% NaCl pH 7.0 (to remove *T. pallidum* remaining in the pellet). The suspension was subjected to a final 400 × *g* centrifugation step for 7 min, after which the resulting supernatant was recovered. The supernatant was then heat-treated at 56°C for 15 min to ensure complete viable *T. pallidum* removal, generating the final IEC sample. A schematic of VTP and IEC sample preparation methods is shown in Supplementary Figure S1. Removal of *T. pallidum* from the IEC sample was confirmed by darkfield microscopy and *T. pallidum*-specific qPCR which showed a 68× reduction in *T. pallidum* DNA. For the CBA experiments, the VTP sample was prepared as outlined above, while the IEC sample was prepared solely by syringe filtering the supernatant.

### 2.3. Endothelial cell culture and sample preparation

For the proteomic experiments, human cerebral brain endothelial cells (hCMEC/d3; Cedarlane, Burlington, ON), also referred to as BECs, were grown in arginine- and lysine-free Dulbecco’s modified Eagle’s medium (Caisson Laboratories, Smithfield, UT) supplemented with human basic fibroblast growth factor (Millipore, Etobicoke, ON), 15% dialyzed fetal bovine serum (dFBS; Gibco, Gaithersburg, MD), and VascuLife EnGS-MV supplements according to the manufacturer’s instructions (Lifeline Cell Technology, Oceanside, CA). Cells were grown in T25 flasks at 37°C in 5% CO_2_ in the presence of either D_4_-lysine (37.5 mg/liter) and ^13^C_6_-arginine (21.75 mg/liter) for medium isotope-labeled cells, or ^13^C_6_^15^N_2_-lysine (38.5 mg/liter) and ^13^C_6_^15^N_4_-arginine (22.25 mg/liter) for heavy isotope-labeled cells (Sigma-Aldrich, Oakville, ON). Cells were passaged a minimum of three times to allow complete incorporation of metabolic labels (Hoedt et al., 2014), transferred to 6-well tissue culture plates (Corning, Corning, NY) and grown to 90–95% confluence. Cell culture supernatant from each well was replaced with 1 mL of either VTP (3.0 × 10^7^) or IEC, with each individual well being considered a biological replicate. To control for any differences between medium or heavy isotope-labeled cells, the SILAC labels were alternated between biological replicates such that two replicates had medium isotope-labeled BECs exposed to VTP or IEC, and two replicates had heavy isotope-labeled BECs exposed to VTP or IEC, for a total of 4 biological replicates per experimental condition. Co-cultures were incubated under microaerophilic conditions (1.5% O_2_, 5% CO_2_, and 93.5% N_2_, at 34°C) for 5 h, cell supernatants were removed, and *T. pallidum* viability was confirmed in the BEC-VTP co-incubation supernatants via darkfield microscopy. BECs were washed 3× with cold saline (0.9% NaCl, pH 7.0), lysed with 50 mM ammonium bicarbonate (NH_4_HCO_3_)/1% (w/v) sodium deoxycholate (DOC) pH 8.0, and heat-treated at 99°C for 5 min to inactivate proteases. Medium and heavy isotope-labeled lysates (300 μg each) were pooled for a total of 600 μg of protein per replicate, the total volume was adjusted to 400 μL using 50 mM ammonium bicarbonate, and samples were reduced with 80 μL of 100 mM dithiothreitol (DTT; 30 min at 37°C), alkylated with 80 μL of 240 mM iodoacetamide (30 min at RT in the dark), and digested with 100 μg of trypsin (1,10 trypsin, protein ratio, 18 h at 37°C). Solid phase extraction cleanup was completed with HLB columns (Waters, MA) using a multiport manifold, proteins were eluted using 300 μL of 60% acetonitrile/0.1% formic acid, and samples were lyophilized and re-suspended in 900 μL of 10 mM ammonium hydroxide pH 10.

For the CBA experiments, BECs were plated at 47,500 cells/well in 24-well tissue culture plates (VWR, Radnor, PA) and grown overnight at 5% CO_2_ in EndoGRO-MV complete culture medium (Millipore). Each well was observed for successful BEC growth prior to incubation at 34°C in a microaerophilic environment of 1.5% O_2_, 5% CO_2_, 93.5% N_2_ with 1 mL of either VTP (5.64 × 10^6^), IEC, or TPCM2 media for 5, 12, 24, and 72 h. Endothelial co-incubations were generally performed in quadruplicate for each timepoint, with each well representing an independent biological replicate, and repeated 3 times with *in vivo T. pallidum* and 2 times with *in vitro T. pallidum*; the exceptions were triplicate *in vivo* VTP samples for the 24- and 72-h timepoints in repeat 1 and the 72-h timepoint in repeat 2. At each timepoint, 180 μL of supernatant was removed from each well, centrifuged at 13,000 × *g* for 5 min, and the resulting cell-free supernatants were stored at −80°C for downstream cytokine analyses. For all samples and timepoints, *T. pallidum* viability was confirmed to be greater than 80% by darkfield microscopy, and BEC viability was confirmed via trypan blue staining.

### 2.4. High pH reversed-phase fractionation

High pH reversed-phase fractionation was completed on an Agilent 1290 HPLC (Agilent, CA) equipped with an Xbridge C18 BEH300 250 mm × 4.6 mm, 5 μm bead size, 300 Å pore size HPLC column (Waters, MA) using 10 mM ammonium hydroxide pH 10 (buffer A) and 80% acetonitrile, 10 mM ammonium hydroxide pH 10 (buffer B). The flow rate was set to 0.75 mL/min, and samples were brought up to a total volume of 0.9 mL with buffer A and injected onto the column. Following a 5-min equilibration period, a gradient of 5–45% buffer B was applied over 75 min, after which fractions were collected every minute for 96 min. Fractions were lyophilized, rehydrated with 200 μL of 2% acetonitrile/0.1% formic acid, and concatenated into 12 fractions by combining every 12^th^ fraction (e.g., fractions 1, 13, 25, 37, 49, 61, 73, and 85 were combined, etc.).

### 2.5. 2D LC–MS/MS workflow and analysis

A 5 μL aliquot of each concatenated fraction was separated by on-line reversed-phase liquid chromatography using a Thermo Scientific EASY-nLC 1,000 system with a reversed-phase pre-column (Michrom Bioresources Magic C18-AQ; 100 μm I.D., 2.5 cm length, 5 μm beads, 100 Å pore size) (Michrom BioResources Inc., Auburn, CA), and an in-house prepared reversed-phase nano-analytical column (Michrom BioResources Magic C-18AQ beads; 75 μm I.D., 15 cm length, 5 μm bead diameter, 100 Å pore size) at a flow rate of 300 nL/min. The chromatography system was coupled on-line with an Orbitrap Fusion Tribrid mass spectrometer (Thermo Fisher Scientific, San Jose, CA) equipped with a Nanospray Flex NG source (Thermo Fisher Scientific). Solvents used were 2% acetonitrile, 0.1% formic acid (Solvent A) and 90% acetonitrile, 0.1% formic acid (Solvent B). Samples were separated using a 120-min gradient (0 min, 5% B; 100 min, 42% B; 15 min, 100% B; 5 min, 100% A). The Orbitrap Fusion Tribrid instrument parameters (Fusion Tune 3.3 software) were as follows: nano-electrospray ion source with spray voltage was 2.55 kV, and capillary temperature was 275°C. Survey MS1 scan m/z range was 350–1,800 m/z in profile mode, resolution was 120,000 FWHM at 200 m/z with one microscan and automatic maximum inject time. The Lock mass for Siloxane 445.120024 m/z was used for internal calibration. Data-dependent acquisition Orbitrap survey spectra were scheduled at least every 3 s, with the software determining the “Automatic” number of MS/MS acquisitions during this period. The automatic gain control (AGC) target values for FTMS (Fourier-transform mass spectrometry) and MSn (multi-stage mass spectrometry) were 400,000 and 10,000, respectively. The most intense ions in the 350–1,800 m/z range with a charge state 2–5 that exceeded 50,000 counts were selected for HCD (higher-energy C-trap disassociation) Iontrap MS/MS fragmentation with detection in centroid mode. Dynamic exclusion settings were as follows: repeat count was set at 2, and exclusion duration was 15 s with a 10 ppm mass window. The ddMS2 IT HCD scan used the following settings: a quadrupole isolation window of 1.6 Da, rapid scan rate, auto mass range, centroid detection, 1 microscan, auto maximum injection time, and stepped HCD collision energy 28, 30, and 32%.

### 2.6. Data analysis and statistical parameters

Raw mass-spectrometry files were created by Xcalibur 4.3.73.11 (Thermo Scientific) and MSFragger v3.2 was used for identification and quantitation. A protein sequence database containing reviewed and non-reviewed entries was downloaded from Uniprot using the identifier for Human (UP000005640; April 7, 2021). Statistical validation for peptides and proteins was determined using the Philosopher toolkit, which uses PeptideProphet and ProteinProphet to filter for high confidence identifications. Both MSFragger (Kong et al., 2017) and Philosopher (da Veiga Leprevost et al., 2020) were accessed through the Fragpipe Graphical User Interface (v.15.0).

Scaffold Q+ (version Scaffold_5.0.1, Proteome Software Inc., Portland, OR) was used for label-based quantitation and peptide and protein identifications. Peptide identifications were accepted if they were established at greater than 95.0% probability by the Percolator posterior error probability calculation (Käll et al., 2008). Protein identifications were accepted if they could be established at greater than 99.0% probability and contained at least 2 identified peptides. Protein probabilities were assigned by the ProteinProphet algorithm (Nesvizhskii et al., 2003). Proteins that contained similar peptides and could not be differentiated based on MS/MS analysis alone were grouped to satisfy the principles of parsimony by Scaffold Q+, while proteins sharing significant peptide evidence were grouped into clusters. Normalization was performed iteratively across samples and spectra by subtracting the average ratios in log-space. Means were used for averaging, and spectra data were log-transformed, pruned of those matched to multiple proteins, and weighted by an adaptive intensity weighting algorithm. Of 996,908 spectra in the experiment at the given thresholds, 75,294 (8%) were included in the quantitation determinations. Differentially expressed (DE) proteins were determined by applying a t-test with a significance level of *p* ≤ 0.05 to all proteins detected and quantified in both medium and heavy labels in at least 2 biological replicates, and a further Benjamini-Hochberg correction (*q* = 0.05) was applied to identify high-confidence proteins. A full list of DE proteins is provided in Supplementary Table S1. Pathway overrepresentation and Gene Ontology (GO) cellular compartment analyses were completed using the ReactomePA package for R (Yu and He, 2016), and the ClusterProfiler R package (Yu et al., 2012), where the *q*-values were set at 0.05 using the Benjamini-Hochberg correction, and pathways or cellular compartments with a corrected *p* value ≤0.05 were considered significant. To identify additional relationships between DE proteins, STRING analysis (V11.5) was completed, and interactions with a combined score > 0.4 were included. Proteins were clustered using the Markov Cluster Algorithm (MCL) with an inflation parameter of 1.5. Analysis of differentially phosphorylated peptides was performed using MSFragger v3.2; phosphopeptides were considered significant if a confidence greater that 95% was attained and detection occurred across both medium and heavy isotopes and in 2 or more biological replicates. Transcription factor analysis was completed using NetworkAnalyst (Zhou et al., 2019) and gene regulatory network transcription factor-gene interactions was performed using the JASPAR (9th release) transcription factor binding site database (Castro-Mondragon et al., 2022). The minimum regulatory transcription factor-gene interaction network included 163 inputted seed DE genes, and a total of 223 nodes with 1,309 edges.

### 2.7. Cytometric bead array and flow cytometry

Cytometric bead array flex sets (BD Biosciences, San Jose, California, USA) were used for detection and quantitation of IL-1α, IL-6, IL-8, VEGF, ICAM-1, IP-10 (interferon gamma-induced protein 10), TNF (tumor necrosis factor), GM-CSF (granulocyte-macrophage colony-stimulating factor), RANTES (regulated upon activation, normal T cell expressed and presumably secreted), and MCP-1. Capture beads and detection antibodies were diluted in FACS buffer (1X PBS + 0.5% bovine serum albumin [Sigma 9 Aldrich, Oakville, ON]) to 1/5 of the manufacturer’s recommended concentration. Cytokine standards (BD Biosciences) were serially diluted two-fold to generate a standard curve for each cytokine from 5,000 pg./mL – 0.625 pg./mL. The upper limit of quantitation (ULOQ) and the lower limit of quantitation (LLOQ) represent the maximum and minimum quantifiable cytokine concentration, respectively. Cell-free supernatant samples from the *T. pallidum*-BEC coincubation were thawed on ice, and 50 μL of supernatant or cytokine standards were incubated in the dark for 1 h at RT with 50 μL of diluted capture beads in 96-well round bottom plates (Corning, Kennebunk, ME), with gentle rocking. Fifty microliters of the detection antibody mix was added to the capture bead-sample mix and incubated for 2 h at RT in the dark, with gentle rocking. The beads were washed twice with 250 μL FACS buffer, plates were centrifuged at 400 × *g* for 3 min at RT using an Eppendorf 5810R centrifuge (Eppendorf Canada, Mississauga, ON), and samples were resuspended in 200 μL of FACS buffer and mean fluorescence intensity (MFI) of each capture bead type was acquired using a CytoFlex Flow Cytometer (Beckman Coulter, Mississauga, ON) using CytExpert software (Beckman Coulter). Cytokine analysis experiments were repeated with four independent biological replicates (defined as individual wells) for each condition, and the assays were repeated three times using *in vivo T. pallidum* from different rabbits, and twice using *in vitro T. pallidum* from separate passages. Cytokine quantitation and statistical analyses were performed for each cytokine using GraphPad Prism 9. One-way ANOVA followed by Dunnetts multiple comparison tests were completed to compare the mean cytokine concentrations of BECs exposed to VTP versus IEC and the basal medium control where *p* values ≤0.05 were considered significant.

### 2.8. DNA extractions and qPCR

Duplicate VTP and IEC samples were centrifuged at 13,226 × *g* (15 min at 4°C) and the resulting pellets were resuspended in 100 μL of lysis buffer (10 mM Tris–HCl, pH 8.0; 0.1 M EDTA; 0.5% SDS) (Lithgow et al., 2021) and DNA extracted using the Qiagen DNeasy Blood and Tissue Kit according to manufacturer’s instructions (Qiagen, Toronto, ON). To normalize extraction efficiency across samples, all samples were spiked with phyB “SPUD” (GenBank ID: Y14572) plasmid DNA as previously described (Lithgow et al., 2021).

*Treponema pallidum* and SPUD DNA were amplified using *flaA*- and SPUD-specific primers, as previously described (Nolan et al., 2006; Lithgow et al., 2021). Averaged Cq values for *flaA* amplification in the VTP and IEC samples were normalized to SPUD, and the reduction in *T. pallidum* DNA in IEC compared to VTP was calculated.

### 2.9. RNA extraction and RT-qPCR

Confluent BECs in 6-well plates were exposed to *in vitro* VTP (3 × 10^7^ cells/well) or equally diluted IEC for 4 h with three individual biological replicates for each treatment. After incubation, cells were washed 3× in cold PBS and incubated in a 1:1 ratio of RNAprotect Cell and RNAprotect Bacteria (Thermo Fisher Scientific) for 10 min on ice. Cells were pelleted at 10,000 × *g* for 10 min, resuspended in 1 mL of RNAprotect, and RNA was extracted using the Qiagen RNeasy kit according to manufacturer instructions, with the addition of an on-column DNA digestion using Qiagen RNase-Free DNase. To prevent RNA degradation, 1 μL of SUPERase-In RNase inhibitor was added to eluted RNA (Thermo Fisher Scientific). cDNA was generated using SuperScript IV First Strand Synthesis (Thermo Fisher Scientific) and RT-qPCR primers for IRF1, TNFAIP2, TRADD, and GAPDH (Panda et al., 2019; Lithgow et al., 2020; Scholz et al., 2020; Liu et al., 2021) were validated to amplify their single target sequence. Successful removal of genomic DNA from samples was confirmed by inclusion of a no-reverse transcriptase control. Data were analyzed using the 2^−ΔΔ*Cq*^ method and normalized to GAPDH mRNA (Livak and Schmittgen, 2001).

### 2.10. Western blotting

Confluent BECs in 6-well plates were exposed to *in vitro* grown VTP (3 × 10^7^ cells/well) or equally diluted IEC for 5 h with 3 biological replicates per treatment condition. Cells were lysed for 30 min on ice with gentle agitation in RIPA lysis buffer containing EDTA-free Protease inhibitor cocktail set 3 (Calbiochem, San Diego, CA) and PhosSTOP phosphatase inhibitor (Roche, Mississauga, ON) according to manufacturer’s instructions. Protein concentration was measured using a Pierce BCA assay (Thermo Fisher Scientific). For western blotting, the 3 biological replicates of BEC lysate from each treatment group (VTP- or IEC-exposed) were pooled, and 13.5 μg pooled whole cell lysate per treatment group was subjected to SDS-PAGE using Bolt 12% acrylamide gels (Thermo Fisher Scientific) and transferred to PVDF membrane (Millipore) via wet-transfer at 400 mA for 2 h, and blocked with Intercept TBS blocking buffer (Licor, Lincoln, NE). Anti-fibronectin (1:1,000) (Sigma-Aldrich, F-3648), anti-MLKL (1:1,000) (Abcam, pS358 EPR9514), and anti-GAPDH (1:1,000) (Cell Signaling Technology, D4C6R) were used as primary antibodies, while the secondary antibodies used were goat anti-rabbit IgG IRDye 800CW (1:20,000) and goat anti-mouse IgG IRDye 680RD (1:20,000) (Licor). Detection and analysis were completed on a Licor Odyssey CLx using Licor Image Studio version 5.2.

## 3. Results

### 3.1. Endothelial cells upregulate secretion of IL-6, IL-8, and VEGF upon *Treponema pallidum* exposure

To assess the inflammatory response of BECs during exposure to *T. pallidum*, we analyzed the supernatant of BEC cultures exposed to *T. pallidum* to measure secretion levels of a panel of 10 cytokines at 5-, 12-, 24-, and 72-h post-exposure using multiplexed CBA assays. To control for confounding effects of rabbit host proteins from either *in vivo* or *in vitro* grown *T. pallidum*, we made comparisons between BECs exposed to viable *T. pallidum* (VTP) and BECs exposed to an infection extract control (IEC) containing no viable *T. pallidum*. We also included cytokine quantification of BECs incubated in basal *T. pallidum* TPCM2 medium to assess basal cytokine levels at each timepoint.

**Figure 1:**
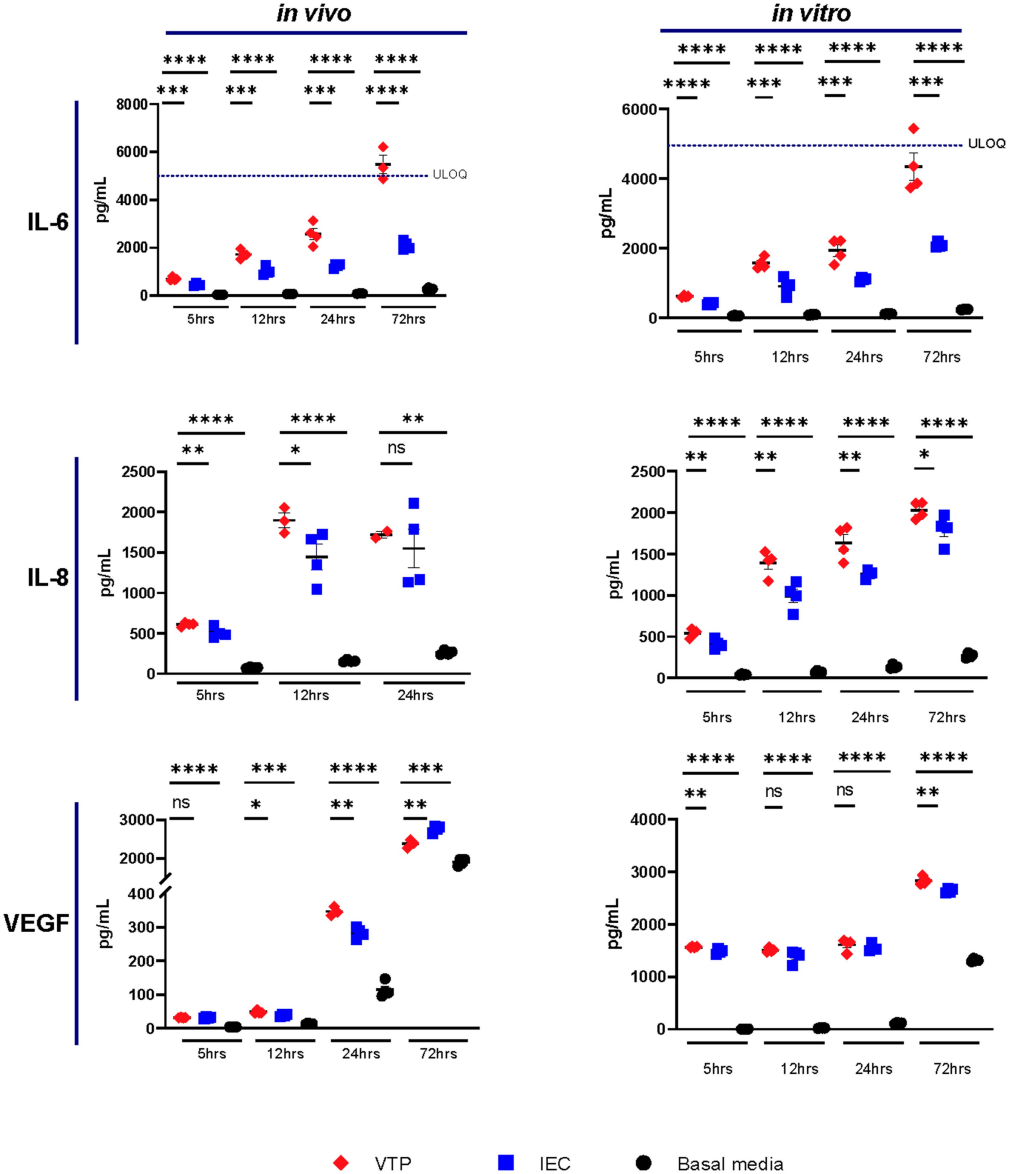
Supernatant concentrations of IL-6, IL-8, and VEGF of BECs exposed to *in vitro-* or *in vivo*-grown *Treponema pallidum* for 5, 12, 24, or 72 h. Data for each cytokine is representative of 3 experimental repeats using *in vivo T. pallidum,* and 2 experimental repeats using *in vitro T. pallidum*. Individual data points represent biological replicates of each experimental condition. Dotted line denotes the upper limit of quantitation (ULOQ). For each timepoint the mean with standard deviation is shown. Statistical analysis was completed using a one-way ANOVA followed by Dunnetts multiple comparison. * *p* < 0.05, **p < 0.01, ***p < 0.001, ****p < 0.0001.

Of the 10 cytokines we analyzed by CBA, we observed increased secretion levels of IL-6, IL-8, and VEGF by BECs exposed to either *in vivo-* or *in vitro*-grown VTP compared to BECs exposed to IEC, and BECs exposed to uninfected basal media (Figure 1; Supplementary Figures S2–S10). Increased levels of IL-6 and IL-8 secretion were consistently observed from BECs exposed to *in vivo-*grown VTP (Figure 1), although the magnitude of secretion levels varied among BECs exposed to VTP isolated from different rabbits (Supplementary Figures S2, S3), an unsurprising finding based upon the varying immune selection pressures in this outbred animal model. Interleukin-6 and IL-8 secretion was also significantly increased in BECs exposed to *in vitro*-grown VTP (Figure 1) at all timepoints and in all experimental repeats (Supplementary Figures S2, S3). BECs exposed to either *in vivo* or *in vitro* IEC consistently secreted higher levels of IL-6 and IL-8 compared to BECs incubated in basal medium, though to a significantly lesser degree than BECs exposed to VTP (Figure 1). We also observed an upward trend in IL-6 secretion by BECs exposed to *in vivo-* or *in vitro-*grown VTP compared to IEC over time, suggesting that IL-6 secretion increases with longer *T. pallidum* exposure (Figure 1). Similarly, we observed increased secretion of VEGF in BECs exposed to *in vivo*- and *in vitro-*grown VTP compared to IEC at most timepoints (Figure 1; Supplementary Figure S4). Interestingly, at the 5-, 12- and 24-h timepoints, VEGF concentrations were 5- to 10-fold higher in BECs exposed to *in vitro*-grown VTP compared to BECs exposed to *in vivo*-grown VTP (Figure 1; Supplementary Figure S4).

Overall, IL-6, IL-8, and VEGF cytokine secretion observed in BECs exposed to IEC was higher than that of BECs exposed to basal media; this observation illustrates that the *T. pallidum* co-culture media itself contains inflammatory components and emphasizes the importance of controlling for these contaminating materials when conducting experiments with *T. pallidum*. Additionally, these data document the consistency of responses raised against *in vitro T. pallidum* populations compared to those raised against *in vivo T. pallidum* isolated from different rabbits.

### 3.2. BECs exposed to *Treponema pallidum* decreases secretion of MCP-1

**Figure 2:**
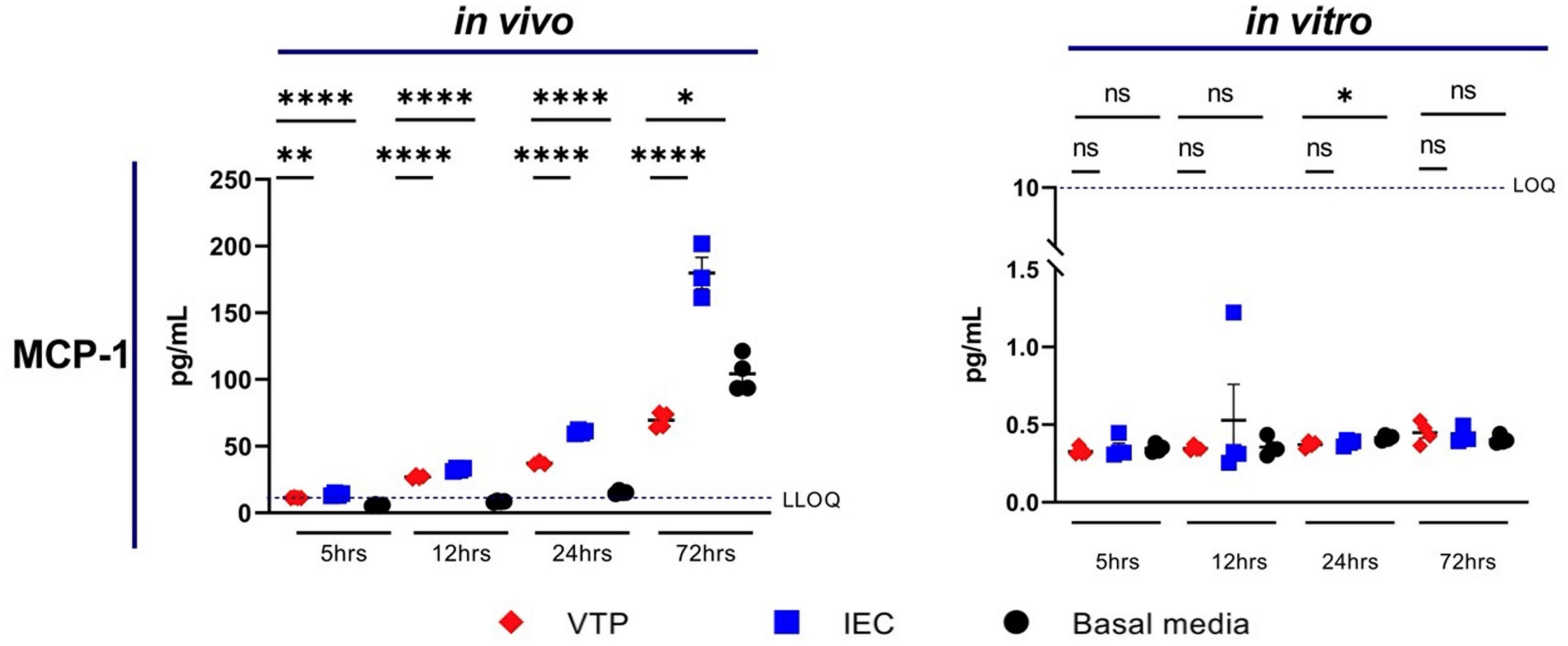
Supernatant concentrations of MCP-1 of BECs exposed to *in vitro* or *in vivo T. pallidum* for 5, 12, 24, and 72 h. Data for each cytokine is representative of 3 repeats using *in vivo T. pallidum,* and 2 repeats using *in vitro T. pallidum.* Dotted line denotes the lower limit of quantitation (LLOQ). For each timepoint the mean with standard deviation is shown. Statistical analysis was completed using a one-way ANOVA followed by Dunnetts multiple comparison. *p < 0.05, **p < 0.01, ***p < 0.001, ****p < 0.0001.

Exposure to *in vivo*-grown VTP, but not *in vitro*-grown VTP, resulted in decreased secretion of MCP-1 from BECs (Figure 2; Supplementary Figure S5). Secreted MCP-1 concentrations were consistently below the lower limit of quantification (LLOQ) after 5 h of *T. pallidum* exposure, suggesting there is a delay in MCP-1 secretion after exposure to *T. pallidum* (Figure 2; Supplementary Figure S5). We were able to measure differences in MCP-1 secretion by BECs exposed to *in vivo T. pallidum* or IEC in two of three experiments, where quantifiable MCP-1 was measured after 12-, 24-, and 72-h of exposure to VTP or IEC, and basal MCP-1 secretion was measurable after 24- and 72-h (Figure 2; Supplementary Figure S5). Attenuation of MCP-1 secretion was most significant at the 24- and 72-h timepoints, where MCP-1 secretion in response to VTP was below that of the basal media control after 72 h (Figure 2; Supplementary Figure S5). Overall, we report that secretion of MCP-1 is reduced in BECs exposed to *in vivo-*grown *T. pallidum,* indicating that viable *T. pallidum* can downregulate secretion of this cytokine during contact with the host endothelium.

### 3.3. Proteomics analysis reveals *Treponema pallidum* exposure alters endothelial protein expression

To expand on the secretomic investigations, we measured global protein expression changes in BECs exposed to *T. pallidum* in comparison to exposure to the IEC control using SILAC and 2D LC–MS/MS. To control for BEC variability between heavy- and medium-labeled BEC, two biological replicates each of heavy- or medium-labeled BECs were exposed to VTP or IEC for 5 h at a multiplicity of infection (MOI) of 30, for a total of 4 biological replicates of pooled heavy and medium cell lysates.

A total of 5,598 proteins were detected in at least one sample with high confidence out of the total possible 13,962 protein coding transcripts present in BECs, corresponding to 41% coverage of the endothelial proteome (Kalari et al., 2016). Of the 5,598 proteins detected, 245 were DE and detected in 2 or more biological replicates. Of the detected DE proteins, 38 had expression changes that were highly significant (below the Benjamini-Hochberg procedure *p* < 0.00116) (Supplementary Table S1). A median Log2 fold-change (Log2FC) of 0.15 for upregulated proteins and −0.25 for downregulated proteins was determined, and a Log2FC cut-off of ± 0.15 was set, generating a pool of 164 proteins where 102 were upregulated and 62 were downregulated in BECs exposed to VTP versus IEC.

### 3.4. Overrepresented pathways and cellular compartments

**Figure 3:**
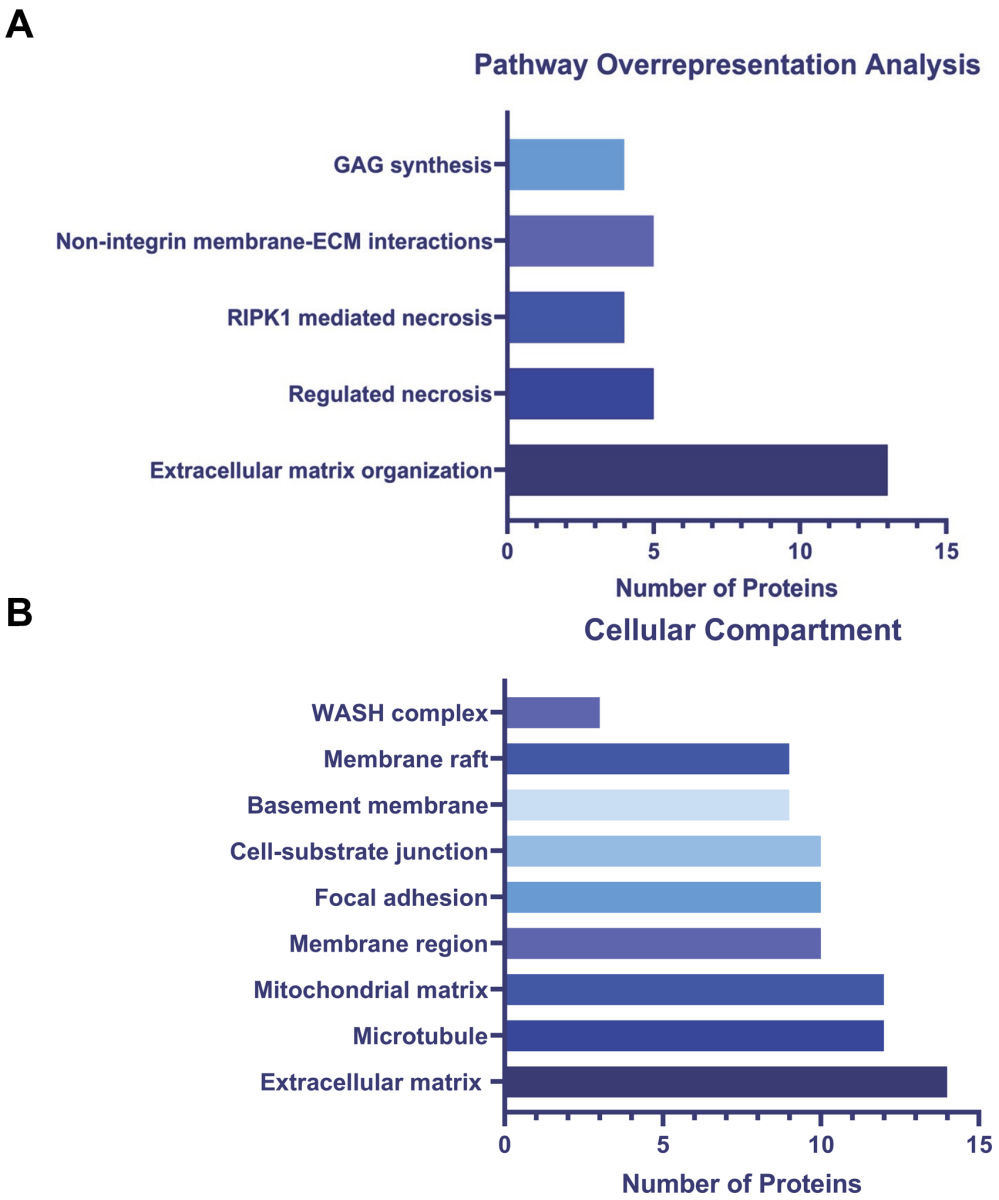
**(A)** ReactomePA overrepresentation analysis of 164 DE proteins. **(B)** ClusterProfiler GO cellular compartment overrepresentation analysis. Pathways and cellular compartments were deemed significant with a Benjamini-Hochberg adjusted *p* value ≤0.05 with a *q*-value of 0.05.

**Figure 4:**
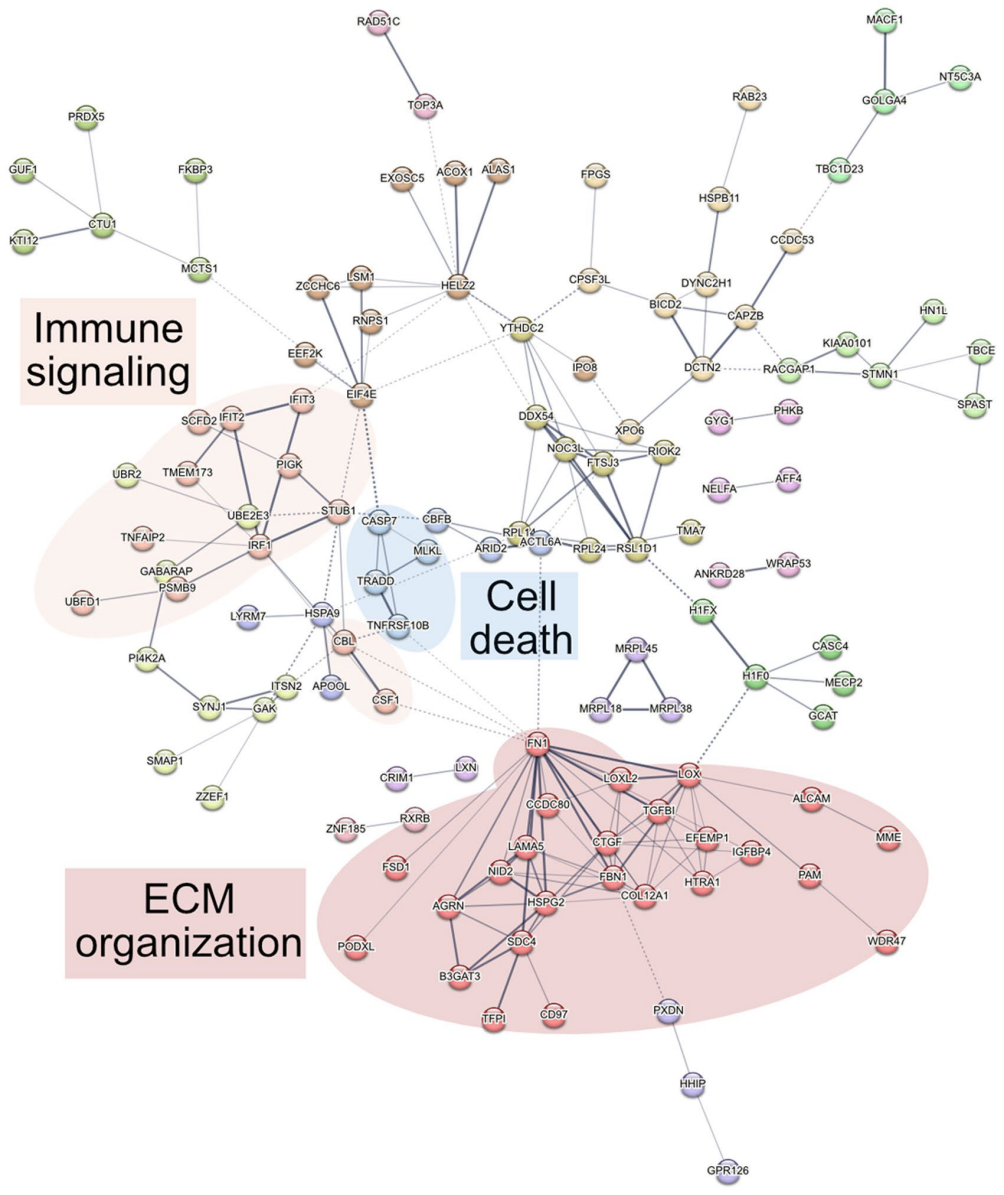
STRING network analysis. Interactions were deemed relevant with a combined score greater than 0.4, and MCL clustering was completed with an inflation parameter of 1.5. 164 nodes were entered which have 200 edges, an average node degree of 2.44, average local clustering coefficient of 0.462, and a PPI enrichment *p* value of 1.84 × 10^−09^. Non-connecting proteins were removed from the figure but included in the analysis.

To determine altered host pathways in BECs exposed to *T. pallidum,* we completed pathway overrepresentation analysis with ReactomePA (Yu and He, 2016), and screened the 164 DE proteins for overrepresented cellular compartments with ClusterProfiler and GO cellular compartment analysis (Yu et al., 2012; Figure 3; Supplementary Tables S2, S3). These analyses determined that our list of DE proteins is enriched for pathways involved in ECM organization, ECM-membrane interactions, and programmed necroptotic cell death (Figure 3A). Notably, our dataset is also significantly enriched for proteins localizing to the WASP and SCAR homolog (WASH) complex, and ECM and membrane regions such as focal adhesions and lipid rafts (Figure 3B). To map protein interactions and to cluster functionally and physically interacting proteins, we analyzed our DE proteins with the STRING database (Figure 4). STRING identified our list of DE proteins as being significantly enriched for protein interactions, with a protein–protein interaction (PPI) *p* value of 1.84 × 10^−09^. Consistent with our Reactome analysis, STRING cluster analysis identified ECM proteins as the largest cluster, followed by pathways involved in immune responses and programmed cell death.

### 3.5. Endothelial ECM composition is altered during *Treponema pallidum* exposure

Reactome pathway overrepresentation identified multiple pathways related to ECM organization, and STRING identified a highly connected cluster of 25 ECM proteins, where each protein is connected to an average of 5 other proteins within the cluster (Figures 4 5). Of the 13 proteins in the ECM organization pathway identified by ReactomePA (Figure 3A; Supplementary Tables S1, S2), all but EFEMP1 (Log2FC 0.21) were decreased in abundance in response to *T. pallidum* exposure. Notably, fibronectin (FN1) was decreased in abundance at −0.69 Log2FC, a finding we validated via western blot in BECs exposed to *in vitro*-grown *T. pallidum* versus an infection extract control (Supplementary Figure S11).

**Figure 5:**
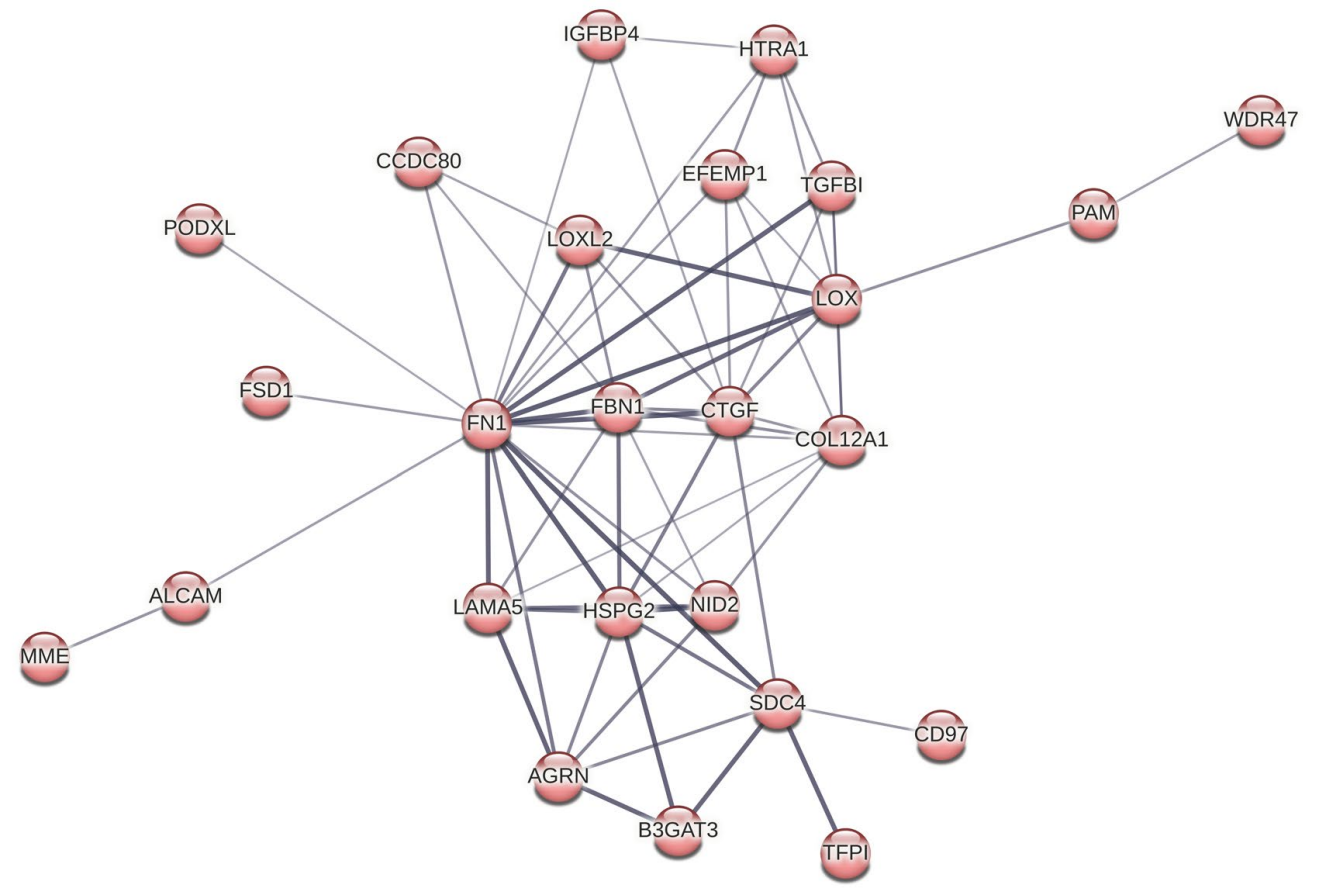
STRING cluster of ECM and cell structure proteins. This cluster has 25 nodes with 64 edges, an average node degree of 5.12, an average clustering coefficient of 0.696, and a PPI enrichment *p* value of <1.0 × 10^−16^.

GO cellular compartment analysis of the 164 DE proteins identified in our proteomics dataset determined that the ECM, membrane regions, cell-substrate junctions, and membrane rafts/microdomains categories were significantly overrepresented (Figure 3B; Supplementary Table S3). Further analysis of the 25 proteins in the STRING ECM and cell structure cluster reveal an enrichment in proteins with epidermal growth factor (EGF)-like domains (NID2, CD97, LAMA5, FBN1, HSPG2, AGRN, EFEMP1), growth factor receptor cysteine-rich superfamily domains (NID2, CD97, IGFBP4, FBN1, CTGF, HTRA1, EFEMP1), and insulin-like growth factor-binding protein (IGFBP) domains (IGFBP4, CTGF, HTRA1), among other similar domains capable of engaging host cell receptors (Supplementary Table S4). ReactomePA overrepresentation analysis of the 25 DE proteins in this STRING cluster identified them as being enriched for proteins with integrin, laminin, and non-integrin membrane-ECM signaling capabilities (Supplementary Table S5). Finally, we observed altered expression of 5 proteins relating to Rho GTPase cycling (ITSN2, DLC1, CAPZB, IQGAP3, RACGAP1, CCDC88A).

Cellular signal transduction and protein regulation commonly occurs via protein phosphorylation. Thus, to complement our global proteomics analysis, we mined our dataset for differentially phosphorylated proteins via MSFragger (Yu et al., 2020) and identified 5 high-confidence proteins with significant differences in phosphorylation between VTP and IEC treatment groups (Table 1). The functional role of these 5 proteins aligns with the overrepresented pathways from our DE protein dataset (Figure 3), in that they are involved in regulating cytoskeletal dynamics, cell death, and immune signaling.

**Table 1:** Phosphorylation post-translational modifications observed in BECs exposed to *T. pallidum* for 5 h.

Endothelial protein	Function	Phosphorylation site(s)	Log2FC protein, *p* value	Log2FC phosphoprotein	Reference
MARCKS	Actin-crosslinking protein, regulates endothelial permeability	S26, S27	0.14, 0.0046	−0.489, −0.497	Estrada-Bernal et al. (2009) and Jin et al. (2012)
HSP90AB1	Chaperone that plays a role in regulating junction permeability, cell survival and apoptosis, tumor angiogenesis, cell signaling	S255	0.01, ns	−0.193	Stühmer et al. (2008) and Colunga Biancatelli et al. (2022)
NCK1	Cytoskeletal dynamics, VEGF and immune signaling	S85	−0.03, ns	0.688	Stoletov et al. (2004) and Alfaidi et al. (2021)
SPAG1	GTPase	S423	−0.03, ns	0.853	Huang et al. (2016)
LARP1	Cytoskeletal-interacting protein	S774	−0.03, ns	−0.687	Burrows et al. (2010)

### 3.6. DE proteins involved in immune responses

Although we did not see any overrepresented immune system pathways via ReactomePA analysis on our set of DE genes, STRING analysis identified a cluster of 12 proteins centered around three Reactome categories, namely interferon alpha/beta signaling, cytokine signaling in immune system, and immune system (Figures 4 6). Core to this cluster is the transcription factor interferon regulatory factor 1 (IRF1) which was downregulated −0.82 Log2FC. Additional interferon DE proteins identified in our study were interferon induced proteins with tetratricopeptide repeats (IFIT) 2, IFIT3, TMEM173 (also known as STING1), and STUB1 (also known as CHIP), which were downregulated. Further, TNF alpha-induced protein 2 (TNFAIP2) was observed to be decreased in expression −1.2 Log2FC (Supplementary Table S1). Using reverse transcriptase quantitative PCR (RT-qPCR), we validated the downregulation of both IRF1 and TNFAIP2 transcripts in BECs exposed to *T. pallidum* versus IEC across 3 biological replicates (Supplementary Figure S12). Finally, consistent with the observed reduction in MCP-1 secretion in our cytokine analysis, macrophage colony stimulating factor 1 (CSF1) was downregulated −0.45 Log2FC in our proteomics analysis.

**Figure 6:**
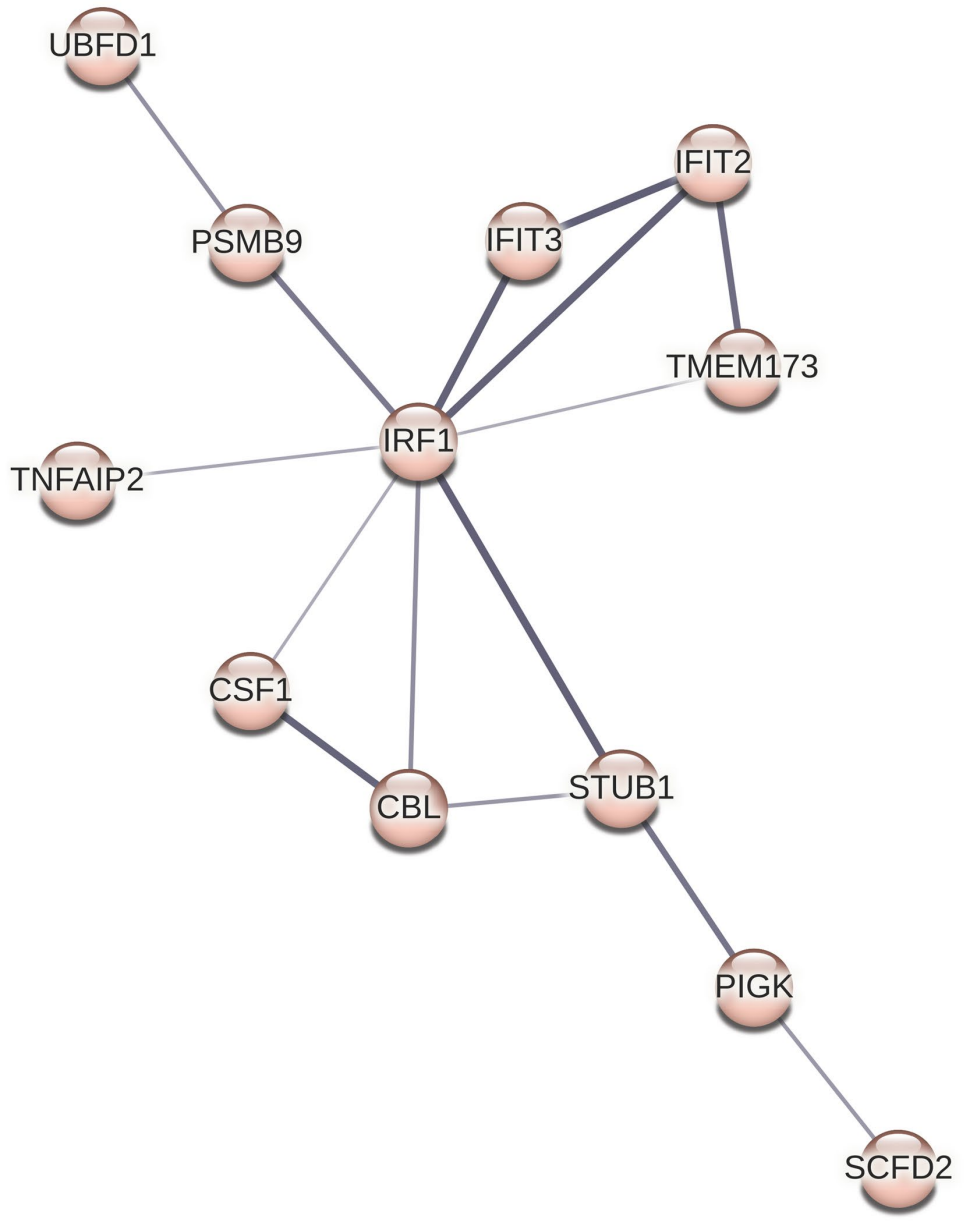
STRING cluster of immune proteins. This cluster contains 12 nodes with 15 edges, an average node degree of 2.5, average local clustering coefficient of 0.651, and a PPI enrichment *p* value of 9.7 × 10^−13^.

### 3.7. DE proteins involved in necroptosis

In our study, ReactomePA pathway overrepresentation analysis identified regulated necrosis and RIPK1 mediated necrosis as overrepresented pathways in BECs exposed to *T. pallidum* (Figure 3A; Supplementary Table S2). Within these pathways, mixed lineage kinase domain-like protein (MLKL) and TNFR1-associated death domain protein (TRADD) were upregulated. Additionally, other apoptotic factors STUB1, TNFRSF10B (also known as death receptor 5 [DR5]), caspase 7 (CASP7) and IRF1 were downregulated. STRING cluster analysis also identified a functionally interrelated cluster of necroptosis proteins consistent with the pathway overrepresentation results, where MLKL, TRADD, TNFRSF10B, and CASP7 were clustered together (Figure 4). BEC viability was assessed during the *T. pallidum*-BEC co-incubations for both the proteomics and secretomics analyses, however, there was no observed increase in cell death in VTP-exposed BECs. We were also unable to detect phosphorylation of MLKL on S358, which would indicate late-stage activation of necroptosis (Sun and Wang, 2014) in *T. pallidum* exposed BEC that were lysed in the presence of phosphatase inhibitors (data not shown). However, we confirmed upregulation of TRADD and downregulation of IRF1 with RT-qPCR in BEC exposed to *T. pallidum*, highlighting the priming of cell death and TNF signaling through TRADD (Supplementary Figure S12).

### 3.8. Gene regulatory network and transcription factor analysis

**Figure 7:**
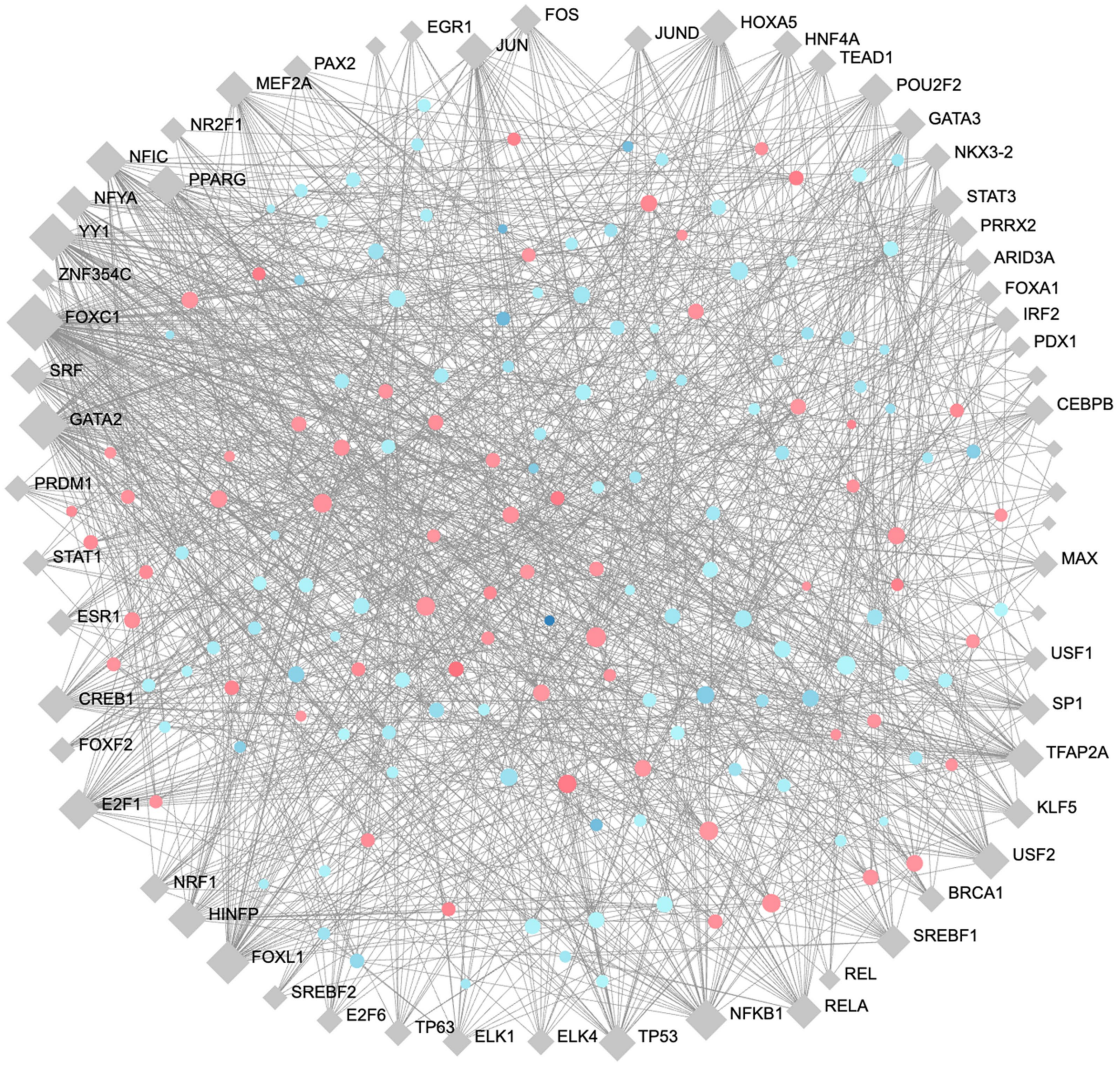
Minimum order transcription factor-gene regulatory network map of DE genes. This network contains 163 seed nodes, and a total of 223 nodes, with 1,309 edges. Transcription factors shown in blue diamonds, upregulated proteins in red circles, and downregulated proteins blue circles. The size of each node represents the number of connections. Figure generated using NetworkAnalyst (Zhou et al., 2019).

To identify high importance transcription factors influencing expression of our DE proteins, we completed gene regulatory network analysis for transcription factor (TF)-gene interactions, using the JASPAR transcription factor database and Network Analyst (Zhou et al., 2019; Castro-Mondragon et al., 2022). A minimum-order TF-Gene network interaction map was generated with 163 seed nodes, 223 total nodes, and 1,309 edges, demonstrating a high-degree of connectedness (Figure 7). The full list of TFs ranked by their degree of connectivity with the DE proteins in our study is listed in Supplementary Table S6. The most highly connected TFs are FOXC1, GATA2, YY1, FOXL1, and NFKB1 with 107, 69, 62, 47, and 42 degrees of connectivity, respectively. Notably, RelA, which is also involved in the formation of the NFKB complex, was identified with 26 degrees of connectivity.

## 4. Discussion

*Treponema pallidum* is a highly invasive bloodborne pathogen that readily traverses the endothelium, including tightly regulated endothelial barriers such as the BBB and placental barrier, to establish a chronic and systemic infection (LaFond and Lukehart, 2006; Salazar et al., 2007). Despite advances in characterizing proteins integral to *T. pallidum* pathogenesis, how *T. pallidum* manipulates the host response to enable transendothelial migration, dissemination, and chronic infection remains incompletely understood. Here we address this knowledge gap by applying immune secretomic profiling and global proteomic analysis of BECs exposed to viable *T. pallidum*.

Via immune secretomic profiling, we demonstrate that *in vitro* cultured BECs exposed to *in vivo* and *in vitro T. pallidum* increase secretion of IL-6, IL-8, and VEGF and decrease secretion of MCP-1 over a time course of 5 to 72 h. Our results are supported by previous research showing increased secretion of IL-6 from human dermal vascular smooth muscle cells (HDVSMCs) exposed to *T. pallidum* (Gao et al., 2019a), and elevated serum concentrations of IL-6 in individuals diagnosed with syphilis (Yan et al., 2017). Increased secretion of IL-6 and promotion of apoptosis was previously observed in bEnd3 brain microvascular endothelial cells exposed to the *T. pallidum* ECM adhesin Tp0751 (Lu et al., 2022). Further, upregulation of IL-6 has been reported in hCMEC/d3 cells exposed to the neuro-invasive pathogens *Neisseria meningitidis* and *Streptococcus suis* to promote bacterial endothelial traversal by disrupting endothelial junction integrity (Vadeboncoeur et al., 2003; Dick et al., 2017). Similarly, our results showing that *T. pallidum*-exposed BECs increase secretion of IL-8 support previous reports demonstrating that viable *T. pallidum*, recombinant *T. pallidum* proteins, or antimicrobial peptides (AMPs) expressed by *T. pallidum*, can increase expression of IL-8 in THP-1 cells, human polymorphonuclear neutrophils (hPMNs), HDVSMCs, and HUVECs (Luo et al., 2018; Gao et al., 2019a; Houston et al., 2022; Wang et al., 2022). Increased secretion of IL-6 and IL-8 can facilitate dissemination of pathogens by promoting vascular permeability through reduced expression of the tight junction proteins claudin, occludin, and VE-cadherin (Paul et al., 2003; Blecharz-Lang et al., 2018). IL-6 also induces expression of VEGF (Adachi et al., 2006), secretion of which was increased in BECs exposed to both *in vitro* and *in vivo* grown *T. pallidum* at most timepoints in our study. Increased secretion of VEGF has been shown to be an important mechanism employed by neuroinvasive strains of *Escherichia coli* and *N. meningitidis* to traverse the BBB by downregulating endothelial junction proteins (Yang et al., 2016; Martins Gomes et al., 2019). Importantly, increased levels of VEGF have been observed in cutaneous lesions of individuals with secondary syphilis (Macaron et al., 2003).

We also observed decreased secretion of MCP-1 in BECs exposed to *in vivo T. pallidum* in our CBA analysis, and similarly we observed downregulation of CSF1 (Log2FC -0.45) in our proteomics analysis. Additionally, our lab has described novel *T. pallidum* AMPs that reduce secretion of MCP-1 from macrophage-differentiated THP-1 cells under pro-inflammatory conditions (Houston et al., 2022). In contrast, it was previously reported that MCP-1 expression is increased in *T. pallidum*-exposed human dermal vascular smooth muscle cells (Gao et al., 2019a). This may be due to the differences between the human cell lines, or the inflammatory effect of host contaminating materials derived from the *in vivo T. pallidum* culture system, as we observed increased secretion of MCP-1 when BECs are exposed to IEC compared to basal media in our cytokine analyses. Accordingly, our data highlights the importance of controlling for contaminating host materials when performing cell-based assays with *T. pallidum*. MCP-1 is an important chemokine for the recruitment of monocytes, and CSF1 is an essential growth factor for monocyte/macrophage differentiation, survival, and proliferation (Deshmane et al., 2009; Sehgal et al., 2021). Effective clearance of *T. pallidum* at sites of infection occurs through the induction of a delayed-type hypersensitivity (DTH) response, which is mediated by the infiltration of CD4^+^ T cells and the activation of macrophages to phagocytose and kill *T. pallidum* (Baker-Zander and Lukehart, 1992; Carlson et al., 2011). Downregulated secretion of MCP-1 and CSF1 in BECs exposed to *T. pallidum* may attenuate the migration of monocytes to sites of *T. pallidum* colonization and allow *T. pallidum* to evade clearance and disseminate during early infection.

In our proteomic studies, STRING analysis identified a cluster of 12 related and interacting immune proteins involved in interferon signaling, cytokine signaling, and defense responses to a virus (Figures 4 6). Central to this cluster are IRF1, IFIT2, IFIT3, and TMEM173 (STING1), which were downregulated. Further, these proteins have been shown to be involved in protecting host cells against HIV infection (Su et al., 2011; Tartour et al., 2014; Nissen et al., 2018). We also observed increased expression of the host DNase TREX1, which enables HIV to evade intracellular detection by degrading HIV DNA during infection (Geijtenbeek, 2010). These observations provide molecular evidence to support the clinical observation of frequent HIV and *T. pallidum* co-infections (Douglas, 2009; Karp et al., 2009).

The most significantly enriched pathways for DE proteins in our analysis, and the largest cluster of proteins to emerge from STRING analysis, are comprised of ECM constituents (Figures 3A 4; Supplementary Tables S1, S2). In our study, most ECM proteins were decreased in BECs exposed to VTP, indicating that the ECM undergoes remodeling during *T. pallidum* contact; further, we observed significant enrichment of proteins involved in glycosaminoglycan (GAG) regulation such as HSPG2, AGRN, and SDC4, which were downregulated −0.5, −0.2, and − 0.41 Log2FC, respectively (Supplementary Tables S1, S2). The observed decrease in ECM proteins may be due to either decreased protein expression or ECM degradation/disruption. Both strategies are commonly employed by other extracellular pathogens to modify endothelial integrity by promoting inflammation through the release of damage associated molecular patterns (DAMPs) and cytokines harbored within the ECM, and by disrupting ECM homeostasis (Watanabe, 2004; Shi et al., 2010; Ruggiero et al., 2013; Quintero et al., 2015; Tomlin and Piccinini, 2018; McQuitty et al., 2020). Though we did not observe differential expression of host matrix metalloproteinases (MMPs) or tissue inhibitors of metalloproteinases (TIMPs), previous work demonstrated that *T. pallidum* interferes with the MMP and TIMP balance in THP-1 cells after 24 h of exposure (Lin et al., 2019). Further, the *T. pallidum* proteins Tp0136 and Tp47 similarly promote MMP/TIMPs imbalance in HDVSMCs (Cai et al., 2022) and HUVECS (Gao et al., 2019b), respectively, and the *T. pallidum* proteins Tp0751 and Tp0750 have been shown to degrade multiple ECM components (Houston et al., 2011, 2014).

In our analyses, we also observed significant enrichment of proteins with epidermal growth factor (EGF)-like, and EGF receptor (EGFR) superfamily domains, which are involved in initiating cell signaling (Supplementary Table S4). Importantly, EGF signaling can repress activity and responses from IRF transcription factors (Kalinowski et al., 2018), which we observed to be downregulated. ECM proteins, including fibronectin, can cluster and activate integrins, which then activate host GTPases such as Rho, Rac, and CDC42, promoting endothelial restructuring which aids bacterial dissemination (Lemichez et al., 2010; Quintero et al., 2015). Notably, there is crosstalk between integrins and EGFRs (Ivaska and Heino, 2010; Maldonado and Hagood, 2021), highlighting the diverse functional role *T. pallidum-*ECM interactions may have during infection. The ability for *T. pallidum* to bind fibronectin and other ECM components is well characterized (Cameron, 2003; Cameron et al., 2004; Brinkman et al., 2008), as is the ability of *T. pallidum* proteins to degrade ECM components (Houston et al., 2011, 2014). Our study supports the hypothesis that *T. pallidum* may cause host cytoskeletal rearrangement and signal transduction by using ECM components as a molecular bridge to activate host cell receptors, or by disrupting ECM homeostasis, which are processes utilized by other bloodborne pathogens to facilitate infection (Lemichez et al., 2010; Quintero et al., 2015; Tomlin and Piccinini, 2018). Further support of this hypothesis is the identified importance of lipid rafts in *T. pallidum* traversal of the endothelium (Lithgow et al., 2021); notably, our dataset is significantly enriched in DE proteins localized to lipid rafts (Figure 3B). We also observed decreased expression of the ECM regulatory proteins lysyl oxidase (LOX) and LOX homolog 2 (LOXL2) by −0.26, and − 0.55 Log2FC, respectively. Dysregulation of LOX alters ECM composition and structure and has been identified as a mechanism of controlling vascular permeability, cell mobility, and invasion (Kagan and Li, 2003; Mammoto et al., 2013). Overall, we observed a decrease in ECM structural and regulatory proteins, indicating that the ECM undergoes remodeling during *T. pallidum* contact, which can broadly influence cell function and promote bacterial dissemination (Tomlin and Piccinini, 2018), highlighting the potential signaling role of the ECM during *T. pallidum* infection.

We also identified necroptotic signaling as a significantly overrepresented pathway (Figure 3A; Supplementary Tables S1, S2). Necroptosis can be activated by TNF, interferon, toll-like receptor (TLR) signaling, and through disruption of host metabolic homeostasis and the generation of reactive oxygen species (Kitur et al., 2016; Dhuriya and Sharma, 2018; Wong Fok Lung et al., 2020; Xia et al., 2020). We observed differential expression of necroptosis-associated factors MLKL, TRADD, STUB1, TNFRSF10B (also known as TRAILR2 and DR5), and IRF1 at 0.28, 0.23, −0.17, −0.27, and −0.82 Log2FC, respectively. MLKL plays a critical role in necroptosis via interaction with the key necroptosis pathway signaling molecules RIPK3 and RIPK1 (Sun and Wang, 2014). TRADD mediates cell fate by functioning as an essential adaptor for TNFR1 and RIPK1 signaling, promotes necroptosis if CASP8 is inhibited or low in abundance, mediates the accumulation of reactive oxygen species, and can initiate necroptosis via a RIPK1-independent mechanism (Grootjans et al., 2017; Wang et al., 2019 Wong Fok Lung et al., 2020). STUB1 is an important negative regulator of necroptosis by controlling degradation of RIPK1 and RIPK3 (Seo et al., 2016). TNFRSF10B is a pro-apoptotic receptor that can signal for both apoptosis and necroptosis (Nahacka et al., 2018). IRF1 plays a diverse role in innate immunity, acting as a transcription factor for type 1 interferon genes and other inflammatory genes following activation by IFN, TNF, and pattern recognition receptor signaling (Feng et al., 2021). In line with the observation that *T. pallidum* interferes with apoptosis through inhibition of caspase activity in PMNs (Wang et al., 2022), we observed a − 0.78 Log2FC decrease in caspase-7 (CASP7). However, we were unable to detect phosphorylation of MLKL on S358, which would indicate complete necroptosis signal activation (Sun et al., 2012), nor did we observe BEC death during our BEC-*T. pallidum* co-incubations. Although we did not observe cell death in our study, the alteration of cell death machinery in *T. pallidum-*exposed cells highlights the potential role of programmed cell death signaling during *T. pallidum* engagement with BECs. Further, the upregulation of TRADD and MLKL alongside the downregulation of the necroptosis inhibitor STUB1 suggests the priming, but not complete activation, of necroptosis in our *in vitro* infection model.

Necrosis-like tissue damage is commonly observed during both primary and tertiary syphilis (Carlson et al., 2011), and has been observed in secondary syphilis (McCready et al., 2011). The tertiary disease manifestations of syphilis, particularly necrotic granulomatous lesions, are similar to the symptoms of leprosy caused by *Mycobacterium leprae*, which have recently been identified to occur as a result of both necroptosis and apoptosis (Rodrigues de Sousa et al., 2022). Thus, there is significant overlap between the clinical symptoms of syphilis and tissue damage etiologically attributed to necroptosis. Though we observe necroptotic priming in our study, it is likely that the necessary conditions to execute necroptosis were not met due to the limited *in vitro* co-culture of BECs and *T. pallidum*. Future work investigating the role of programmed cell death during *T. pallidum* infection will provide insight into the disease symptoms that arise during syphilis.

We also completed JASPAR transcription factor enrichment analysis in Network Analyst to identify transcription factors that influence the expression of DE proteins in our proteomics dataset (Zhou et al., 2019; Castro-Mondragon et al., 2022; Figure 7; Supplementary Table S6). Many of the transcription factors identified are involved in inflammation, endothelial permeability, cell fate, and endothelial responses to bacterial infection, including NFKB1, RELA, TP53 (Barabutis et al., 2018), FOS and JUN which form the AP-1 complex (Wang et al., 1999; Liu et al., 2015), EGR1 (de Klerk et al., 2017), and PPARγ (Günzel and Yu, 2013). EGR1 is commonly induced in host cells during bacterial infection through EGFR-ERK1/2 and integrin signaling pathways (de Klerk et al., 2017), and through VEGF (Sassa et al., 2002). We observed upregulation of VEGF and differential expression of integrin- and EGFR-binding ECM proteins in *T. pallidum-*exposed endothelial cells, suggesting the activity of important transcription factors are altered during *T. pallidum* exposure. Additionally, EGR1 and VEGF activity upregulates the transcription factor Snail, which in turn downregulates the expression of tight junction proteins and is involved in the traversal of neuropathogenic bacteria across the BBB (Günzel and Yu, 2013; Kim et al., 2015; Yang et al., 2016; Martins Gomes et al., 2019). Further, EGR1 expression controls IL-8 expression and secretion (Ma et al., 2009), and IL-8 was secreted by *T. pallidum-*exposed BECs in our study. These data implicate the transcription factors EGR1, SNAIL1, AP-1, and PPARγ in the remodeling of endothelial junctions and cytokine expression during *T. pallidum* exposure, warranting future investigation into their potential role during *T. pallidum* dissemination and syphilis disease progression.

## 5. Limitations of this study

Our study is a novel investigation of endothelial responses during *T. pallidum* infection, however, there are limitations to our approach. First, our cytokine analysis demonstrated that the IEC used in our proteomics and immune secretomic analysis is more inflammatory than basal media, leading to comparisons with a treatment group experiencing moderate inflammatory stimulation. This observation highlights the importance of accounting for inflammatory and non-treponemal material originating from both *in vitro* and *in vivo T. pallidum* culture methods. Further, these results corroborate the report that 0.22 μm filtered *T. pallidum* cultures contain immune and inflammatory mediators, but are non-infectious (Huang et al., 2023). Second, we observed differences in selected cytokine secretion profiles (MCP-1 and VEGF) between BECs exposed to *in vivo* versus *in vitro* cultured *T. pallidum*, which likely resulted from differences in the *T. pallidum* culture methods. Third, our proteomics analysis includes a single timepoint and may not capture the transient and dynamic nature of cellular signal transduction. Fourth, this study used *in vitro* grown endothelial cells and may not perfectly recapitulate the molecular responses of endothelial cells to *T. pallidum in vivo*. Finally, the threshold cut-off value selected for determining the DE proteins in *T. pallidum*-exposed BECs (± 0.15) may be considered a limitation of the study. However, it is not expected that large changes in DE proteins would be observed in this study due to the duration of *T. pallidum* exposure, and the nature of *T. pallidum* as a stealth pathogen.

## 6. Conclusion

Here we aimed to map both the cytokine signaling profile and the cellular molecular responses of human microvascular brain endothelial cells to *T. pallidum* exposure. To our knowledge, this is the first study to apply quantitative proteomics and cytometric bead arrays to reveal the proteomic and temporal immune secretomic responses of BECs to *T. pallidum*. We identified ECM composition and regulation as central, differentially regulated processes during *T. pallidum* exposure to the endothelium. Thus, we propose that *T. pallidum-*ECM interactions and ECM dysregulation are important for endothelial signaling during *T. pallidum* exposure, notably due to the previously described finding of *T. pallidum*’s propensity for adherence to the ECM (Cameron, 2003; Cameron et al., 2004; Brinkman et al., 2008). Additionally, this study provides novel molecular evidence that the observed necrotic tissue damage during *T. pallidum* infection may be due to regulated necroptosis. We also identify the transcription factors EGR1, SNAIL1, AP-1, and PPARγ as potential regulators of endothelial immune signaling and junctional organization upon *T. pallidum* contact. We describe increased secretion of the cytokines IL-6, IL-8, and VEGF during *T. pallidum* infection, all of which promote transendothelial migration of pathogens by increasing vascular permeability through inflammation and reduced expression of tight junction proteins (Paul et al., 2003; Günzel and Yu, 2013; Yang et al., 2016; Blecharz-Lang et al., 2018). Additionally, we observed downregulation of MCP-1 and CSF1 which are critical in recruiting, differentiating, and expanding monocytes at sites of infection (Deshmane et al., 2009; Stanley and Chitu, 2014). As macrophages are the primary cell type associated with *T. pallidum* clearance (Baker-Zander and Lukehart, 1992; Carlson et al., 2011), the dampening of monocyte recruitment and macrophage activity would promote *T. pallidum* survival. Thus, we propose that *T. pallidum* engagement with the endothelium dysregulates ECM homeostasis, primes programmed cell death and necroptotic signaling, and induces an inflammatory immune environment which promotes endothelial junction loosening, while attenuating monocyte recruitment and activity. This work informs our understanding of *T. pallidum* pathogenic mechanisms and identifies cellular and immune processes that are central to a successful *T. pallidum* infection.

## Data availability statement

The mass spectrometry raw datasets presented in this study can be found in the MassIVE repository at https://massive.ucsd.edu/MSV000092275/ under the identifier PXD043317.

## Ethics statement

Ethical approval was not required for the studies on humans in accordance with the local legislation and institutional requirements because only commercially available established cell lines were used. The animal study was approved by the Animal Ethics Committee at the University of Victoria. The study was conducted in accordance with the local legislation and institutional requirements.

## Author contributions

SW: Conceptualization, Formal analysis, Writing – original draft, Writing – review & editing, Data curation, Investigation, Methodology. AR: Data curation, Formal analysis, Investigation, Writing – review & editing. AG: Investigation, Writing – review & editing, Methodology. SH: Methodology, Writing – review & editing, Data curation. KL: Writing – review & editing, Conceptualization. AE: Writing – review & editing, Methodology. JF: Investigation, Writing – review & editing, Methodology. KC: Investigation, Writing – review & editing, Methodology. LR: Investigation, Writing – review & editing, Conceptualization, Formal analysis. CC: Conceptualization, Formal analysis, Writing – review & editing, Funding acquisition, Project administration, Supervision, Writing – original draft.

## Funding

This work was supported by grants R37AI051334 and U19AI144133 from the National Institutes of Health (CC) and a Canadian Institutes of Health CGSD Scholarship (SW).

## Conflict of interest

The authors declare that the research was conducted in the absence of any commercial or financial relationships that could be construed as a potential conflict of interest.

## Publisher’s note

All claims expressed in this article are solely those of the authors and do not necessarily represent those of their affiliated organizations, or those of the publisher, the editors and the reviewers. Any product that may be evaluated in this article, or claim that may be made by its manufacturer, is not guaranteed or endorsed by the publisher.
