# Supplementary material for: Syphilis and the host: multi-omic analysis of host cellular responses to Treponema pallidum provides novel insight into syphilis pathogenesis
Source: Front Microbiol. 2023 Sep 19;14:1254342. doi: 10.3389/fmicb.2023.1254342 (PMC10546344; doi:10.3389/fmicb.2023.1254342)
Supplement: Supplementary file 1 [file Data_Sheet_1.pdf]

# *Supplementary Material*

**Syphilis and the host: multi-omic analysis of host cellular responses to *Treponema pallidum* provides novel insight into syphilis pathogenesis**

**Sean Waugh[1], Akash Ranasinghe[1], Alloysius Gomez[1], Simon Houston[1], Karen V. Lithgow[1], Azad Eshghi[2], Jenna Fleetwood[1], Kate M.E. Conway[1], Lisa A. Reynolds[1], Caroline E. Cameron[1,3]***

*Correspondence: Caroline Cameron caroc@uvic.ca

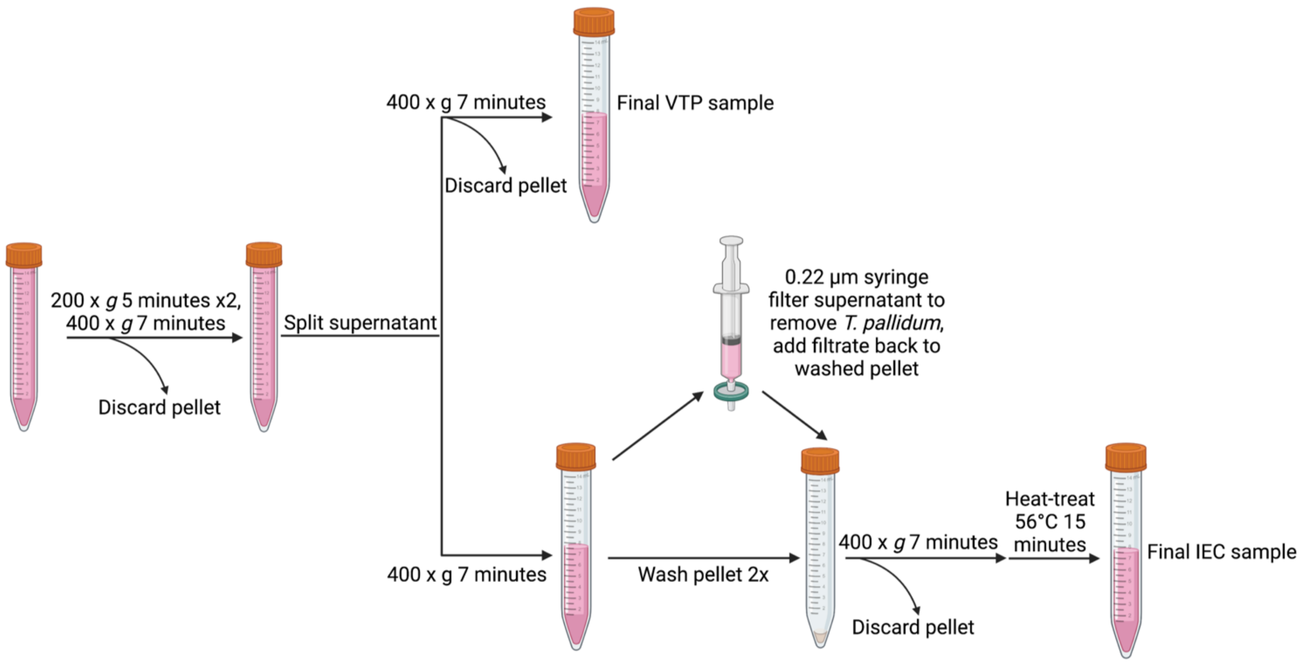


**Supplementary Figure 1.** Preparation of viable *T. pallidum* (VTP) and infection extract control (IEC) samples for endothelial coincubation experiments for proteomic analyses.

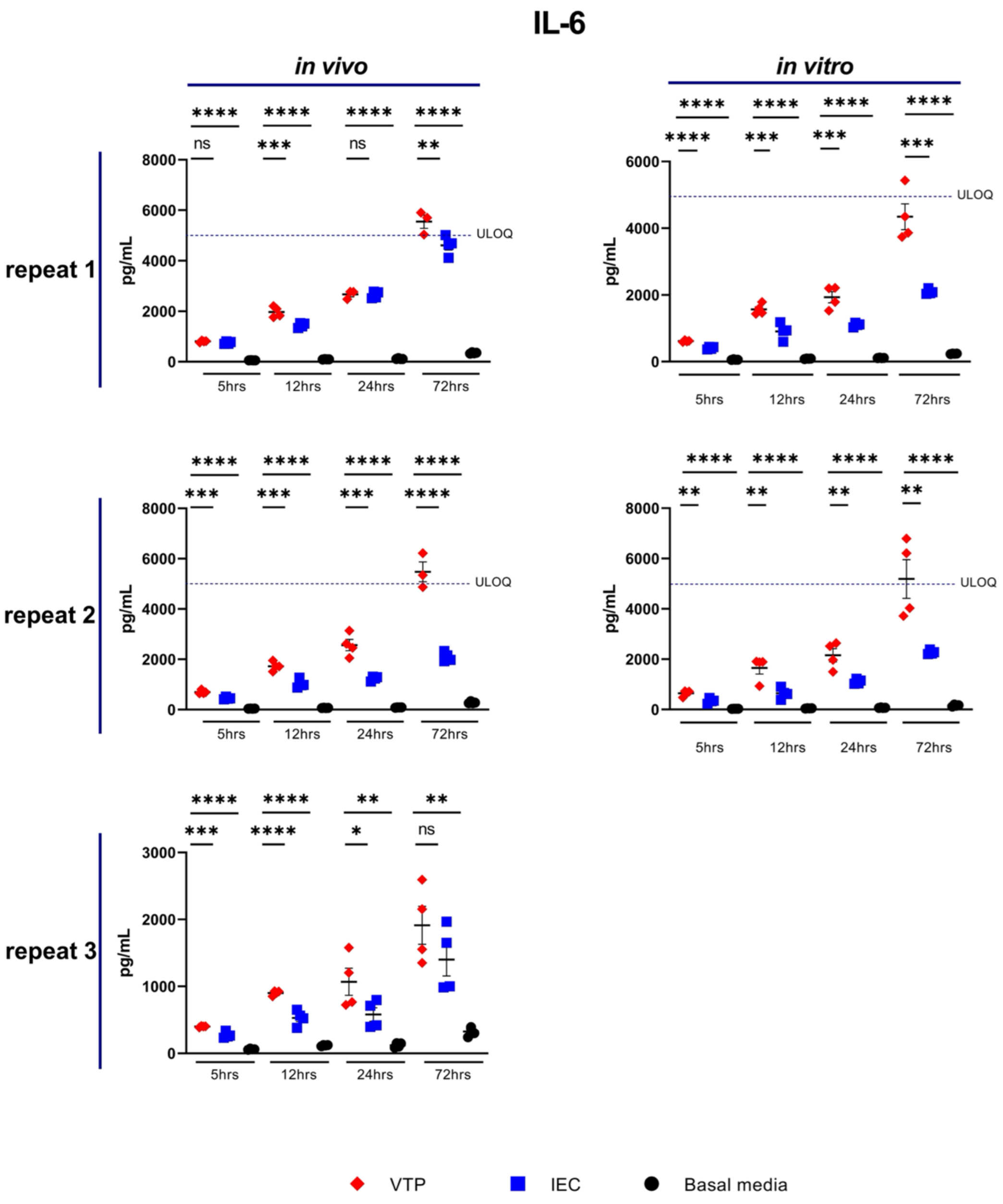


**Supplementary Figure 2.** Concentrations (pg/mL) of IL-6 secreted by hCMEC/d3 cells exposed to viable *T. pallidum* from either the *in vivo* or *in vitro* culture systems. The infection extract control and basal media were included as controls. Culture supernatant was sampled at various timepoints, and secreted cytokines were quantified using a multiplexed cytometric bead array. A representative repeat for *in vivo* and *in vitro* experiments was shown in Figure 1. The upper limit of quantitation (ULOQ) was 5000pg/mL. Statistical analysis was completed using one-way ANOVA followed by Dunnetts multiple comparison. Significant differences are represented as follows: * = p ≤0.05, ** = p ≤0.01, *** = p ≤0.001, **** = p <0.0001, ns = not significant.

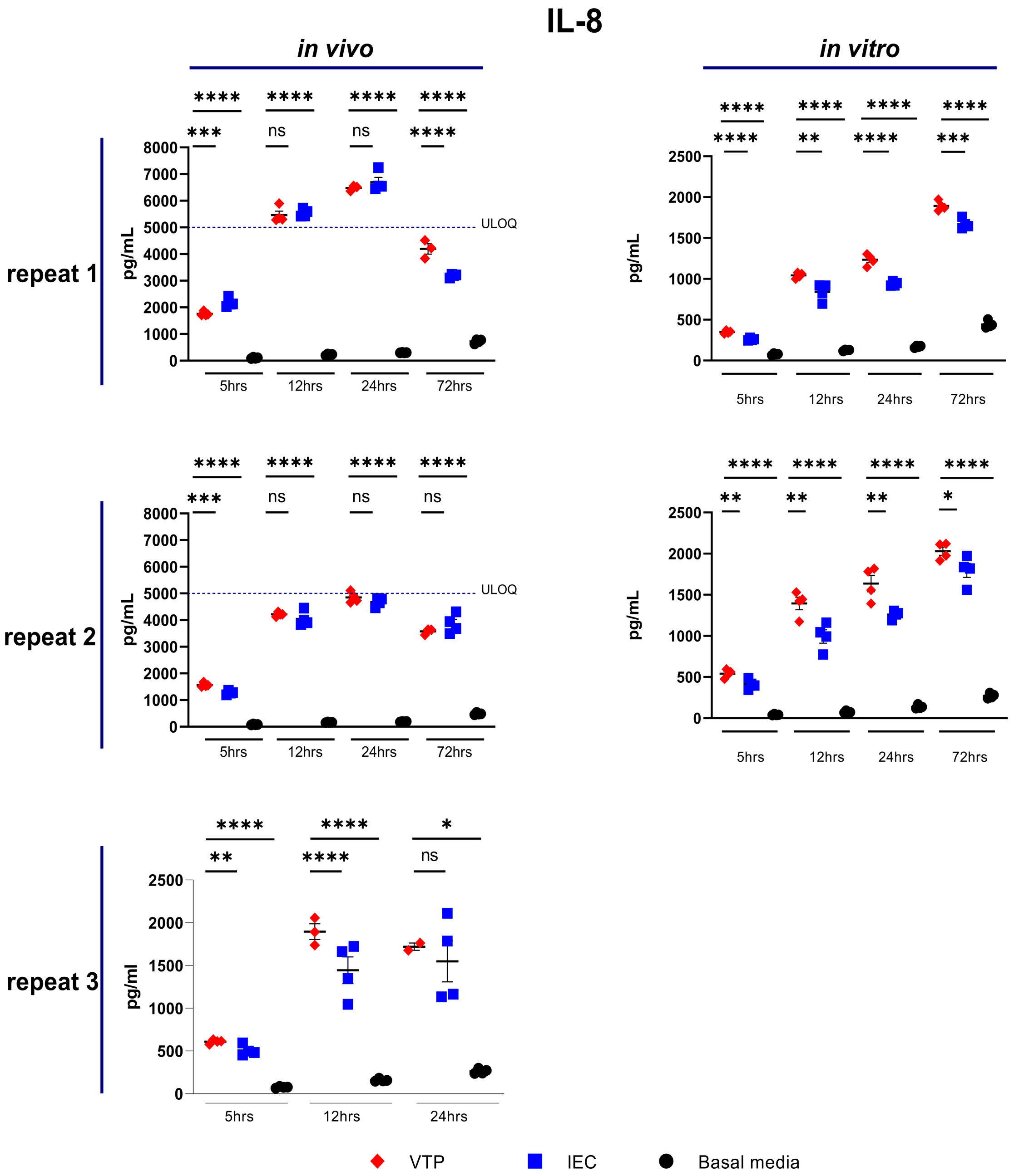


**Supplementary Figure 3.** Concentrations (pg/mL) of IL-8 secreted by hCMEC/d3 cells exposed to viable *T. pallidum* from either the *in vivo* or *in vitro* culture systems. The infection extract control and basal media were included as controls. Culture supernatant was sampled at various timepoints, and secreted cytokines were quantified using a multiplexed cytometric bead array. A representative repeat for *in vivo* and *in vitro* experiments was shown in Figure 1. The upper limit of quantitation (ULOQ) was 5000pg/mL; however, for *in vivo* repeat 3, two VTP biological replicates at 24hrs and one at 12hrs, as well as all VTP and IEC samples at the 72 hour timepoint, were above the ULOQ and were therefore excluded from the analysis. Statistical analysis was completed using one-way ANOVA followed by Dunnetts multiple comparison. Significant differences are represented as follows: * = p ≤0.05, ** = p ≤0.01, *** = p ≤0.001, **** = p <0.0001, ns = not significant.

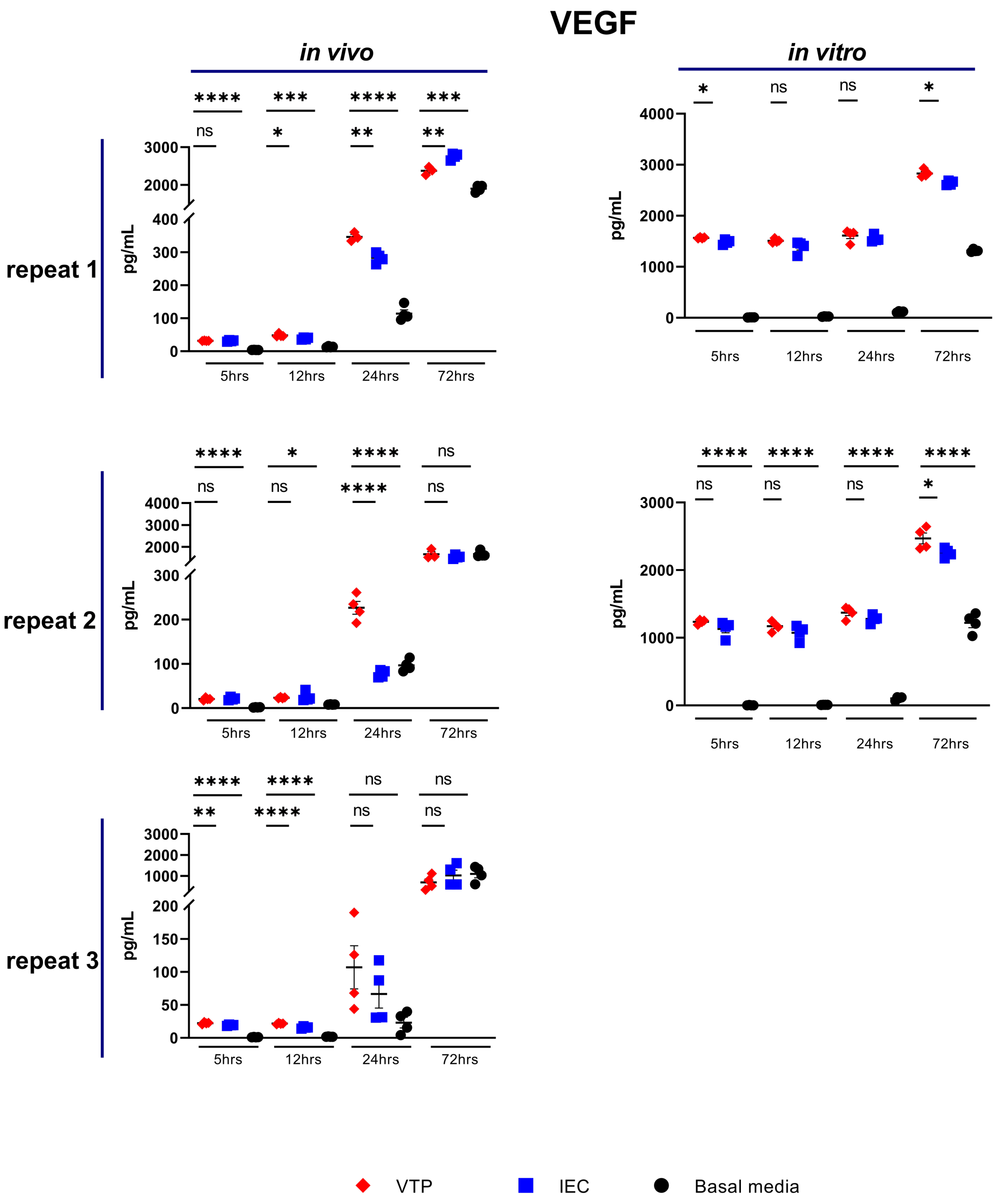


**Supplementary Figure 4**. Concentrations (pg/mL) of VEGF secreted by hCMEC/d3 cells exposed to viable *T. pallidum* from either the *in vivo* or *in vitro* culture systems. The infection extract control and basal media were included as controls. Culture supernatant was sampled at various timepoints, and secreted cytokines were quantified using a multiplexed cytometric bead array. A representative repeat for *in vivo* and *in vitro* experiments was shown in Figure 1. Statistical analysis was completed using one-way ANOVA followed by Dunnetts multiple comparison. VEGF *in vitro* repeat 2 at the 72-hour timepoint comparing viable *T. pallidum* and infection extract control was not significant via Dunnetts multiple comparisons, but was via one-way ANOVA, which we have shown on the figure. Significant differences are represented as follows: * = p ≤0.05, ** = p ≤0.01, *** = p ≤0.001, **** = p <0.0001, ns = not significant.

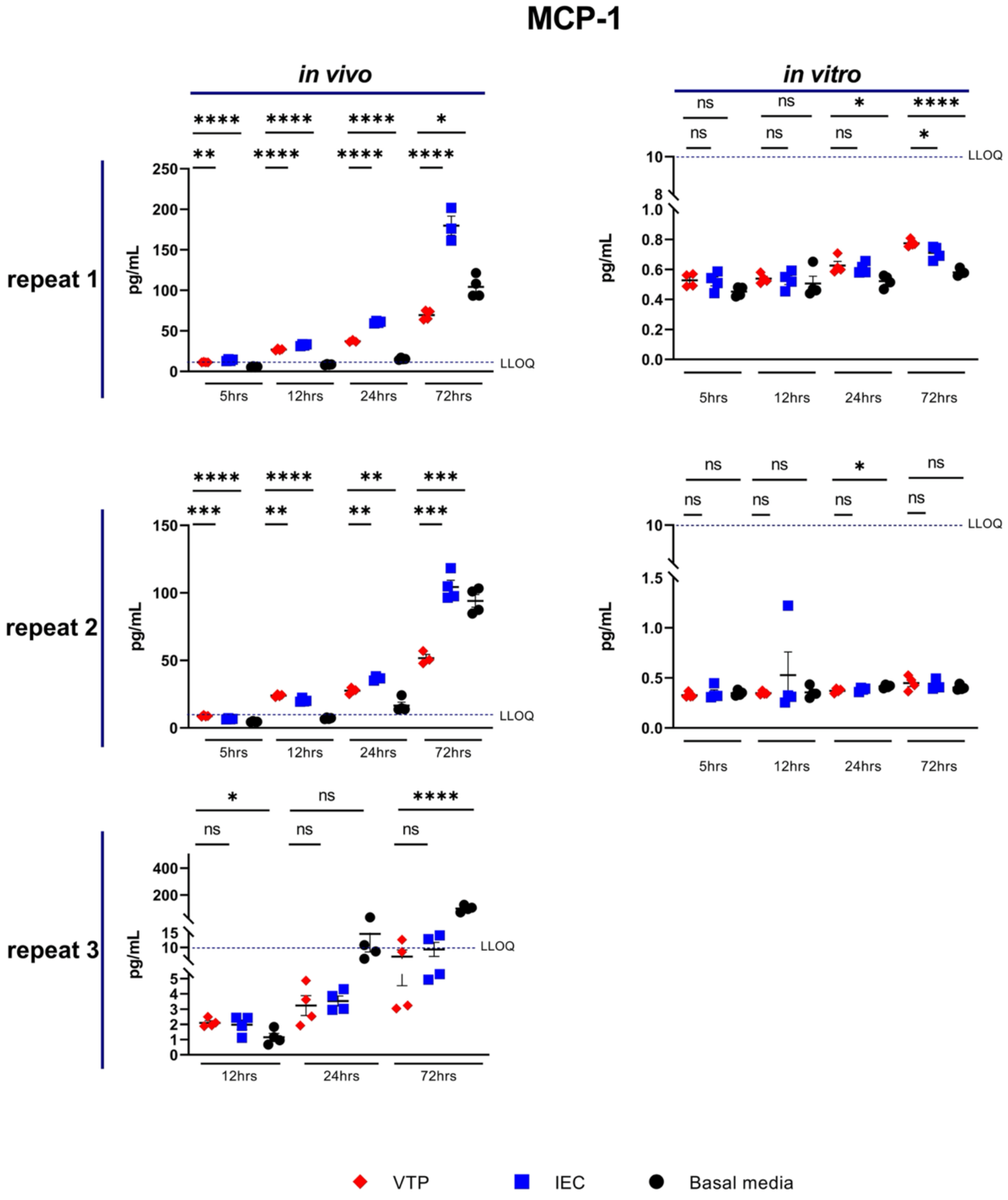


**Supplementary Figure 5.** Concentrations (pg/mL) of MCP-1 secreted by hCMEC/d3 cells exposed to viable *T. pallidum* from either the *in vivo* or *in vitro* culture systems. The infection extract control and basal media were included as controls. Culture supernatant was sampled at various timepoints, and secreted cytokines were quantified using a multiplexed cytometric bead array. A representative repeat for *in vivo* and *in vitro* experiments was shown in Figure 2. The upper limit of quantitation (ULOQ) was 5000pg/mL. Statistical analysis was completed using one-way ANOVA followed by Dunnetts multiple comparison. Significant differences are represented as follows: * = p ≤0.05, ** = p ≤0.01, *** = p ≤0.001, **** = p <0.0001, ns = not significant.

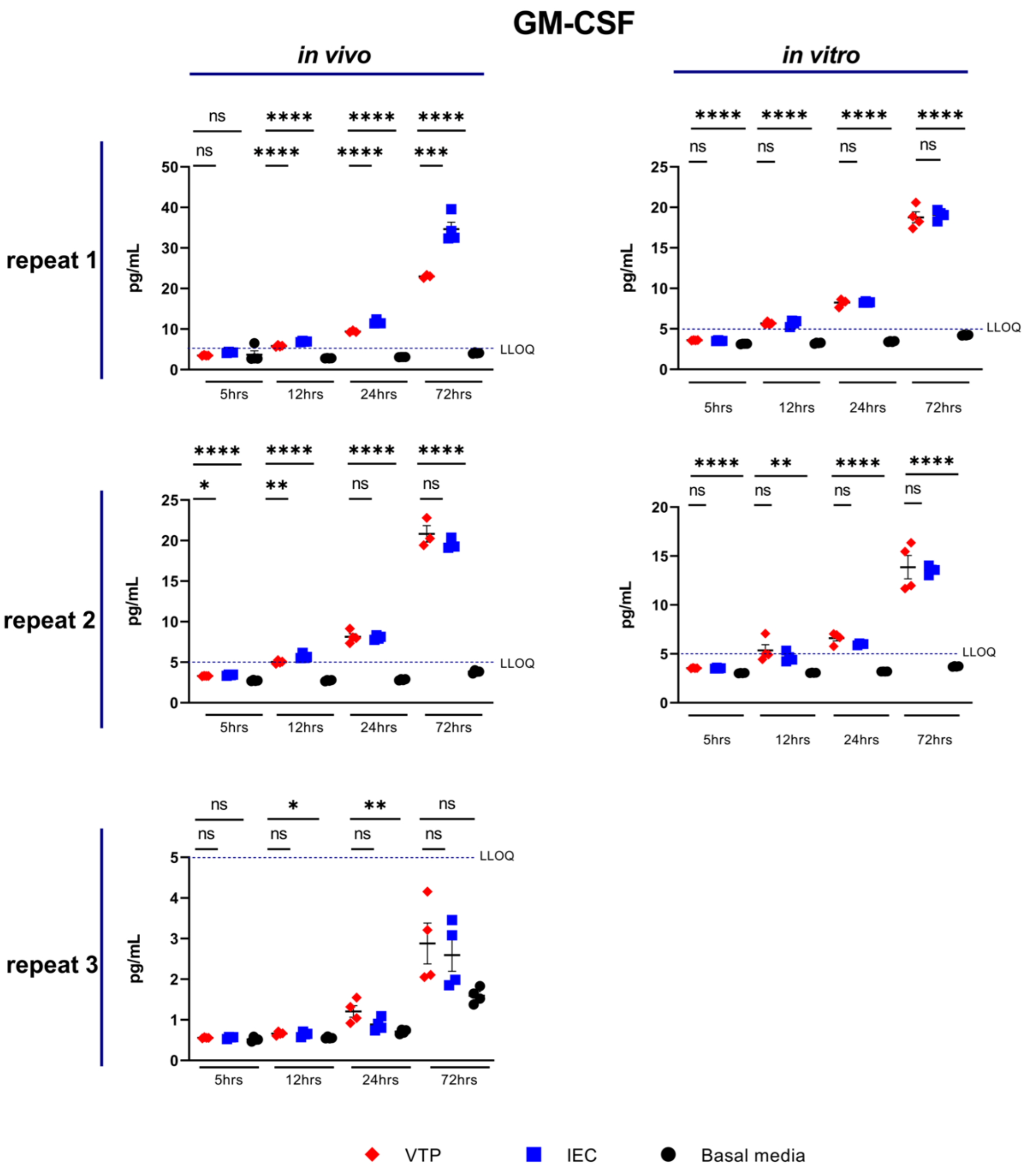


**Supplementary Figure 6.** Concentrations (pg/mL) of GM-CSF secreted by hCMEC/d3 cells exposed to viable *T. pallidum* from either the *in vivo* or *in vitro* culture systems. The infection extract control and basal media were included as controls. Culture supernatant was sampled at various timepoints, and secreted cytokines were quantified using a multiplexed cytometric bead array. The upper limit of quantitation (ULOQ) was 5000 pg/mL. Statistical analysis was completed using one-way ANOVA followed by Dunnetts multiple comparison. Significant differences are represented as follows: * = p ≤0.05, ** = p ≤0.01, *** = p ≤0.001, **** = p <0.0001, ns = not significant.

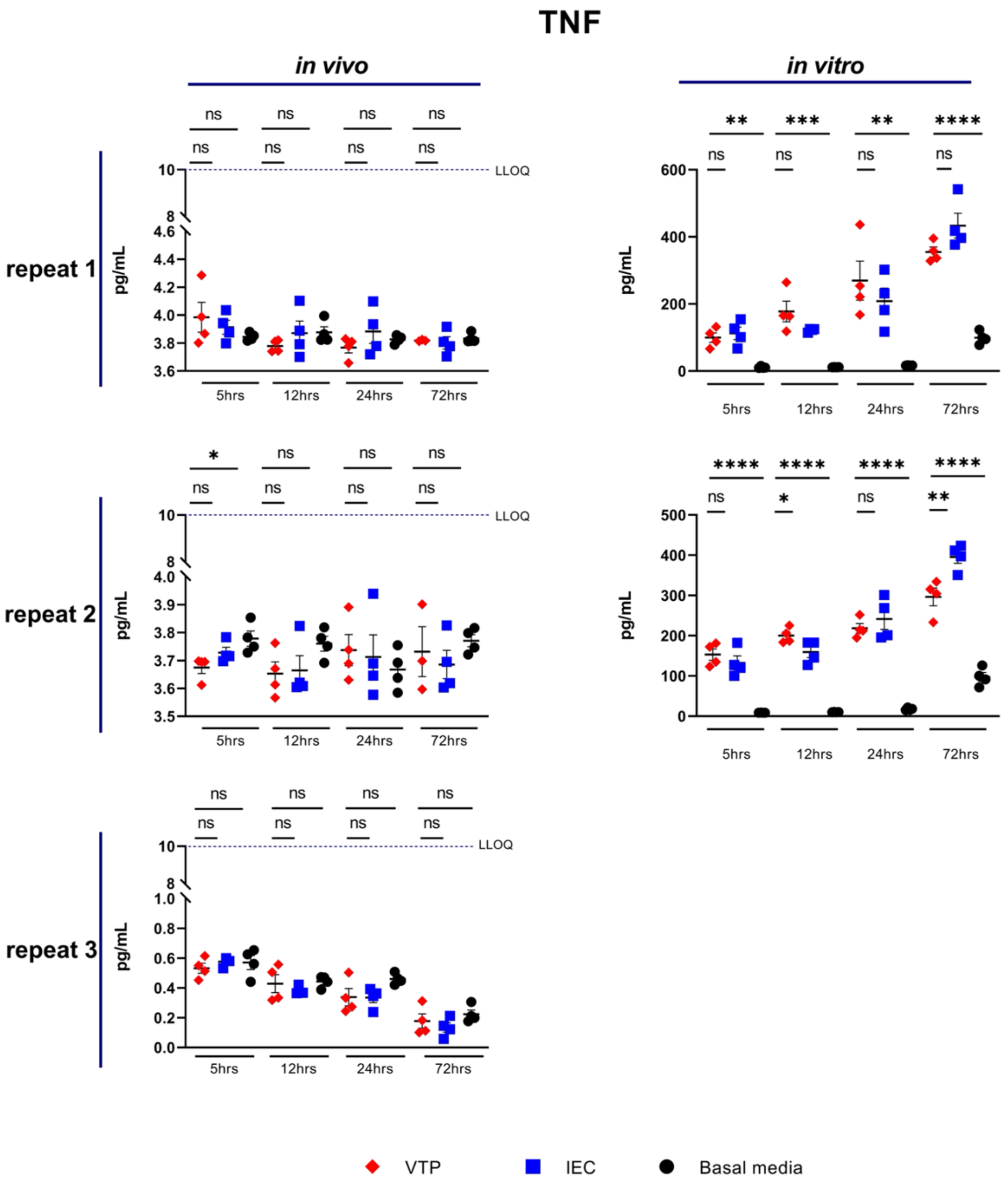


**Supplementary Figure 7.** Concentrations (pg/mL) of TNF secreted by hCMEC/d3 cells exposed to viable *T. pallidum* from either the *in vivo* or *in vitro* culture systems. The infection extract control and basal media were included as controls. Culture supernatant was sampled at various timepoints, and secreted cytokines were quantified using a multiplexed cytometric bead array. The upper limit of quantitation (ULOQ) was 5000 pg/mL. Statistical analysis was completed using one-way ANOVA followed by Dunnetts multiple comparison. Significant differences are represented as follows: * = p ≤0.05, ** = p ≤0.01, *** = p ≤0.001, **** = p <0.0001, ns = not significant.

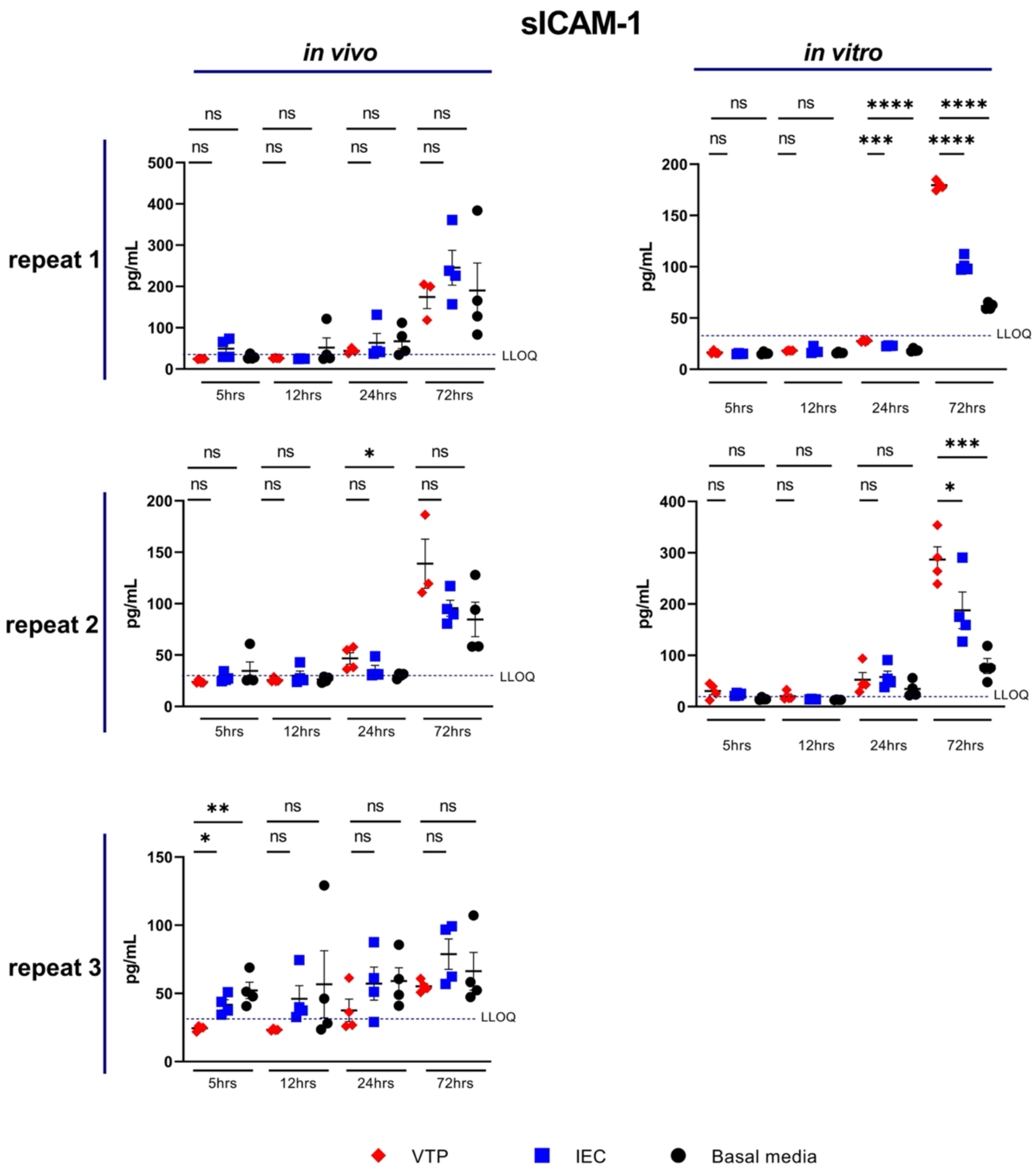


**Supplementary Figure 8.** Concentrations (pg/mL) of soluble ICAM-1 (sICAM-1) secreted by hCMEC/d3 cells exposed to viable *T. pallidum* from either the *in vivo* or *in vitro* culture systems. The infection extract control and basal media were included as controls. Culture supernatant was sampled at various timepoints, and secreted cytokines were quantified using a multiplexed cytometric bead array. The upper limit of quantitation (ULOQ) was 5000 pg/mL. Statistical analysis was completed using one-way ANOVA followed by Dunnetts multiple comparison. Significant differences are represented as follows: * = p ≤0.05, ** = p ≤0.01, *** = p ≤0.001, **** = p <0.0001, ns = not significant.

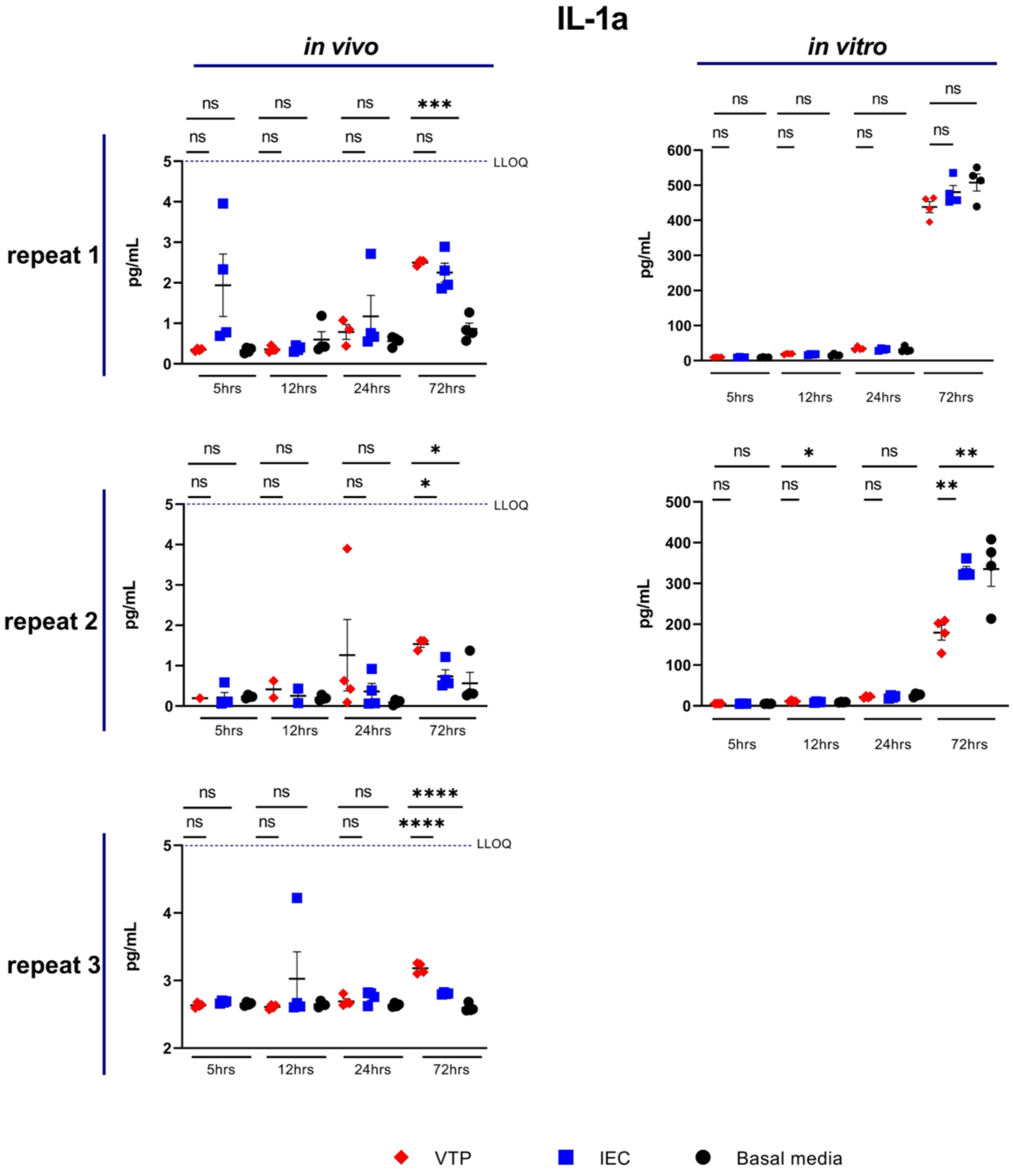


**Supplementary Figure 9.** Concentrations (pg/mL) of IL-1a secreted by hCMEC/d3 cells exposed to viable *T. pallidum* from either the *in vivo* or *in vitro* culture systems. The infection extract control and basal media were included as controls. Culture supernatant was sampled at various timepoints, and secreted cytokines were quantified using a multiplexed cytometric bead array. The upper limit of quantitation (ULOQ) was 5000 pg/mL. Statistical analysis was completed using one-way ANOVA followed by Dunnetts multiple comparison. Significant differences are represented as follows: * = p ≤0.05, ** = p ≤0.01, *** = p ≤0.001, **** = p <0.0001, ns = not significant.

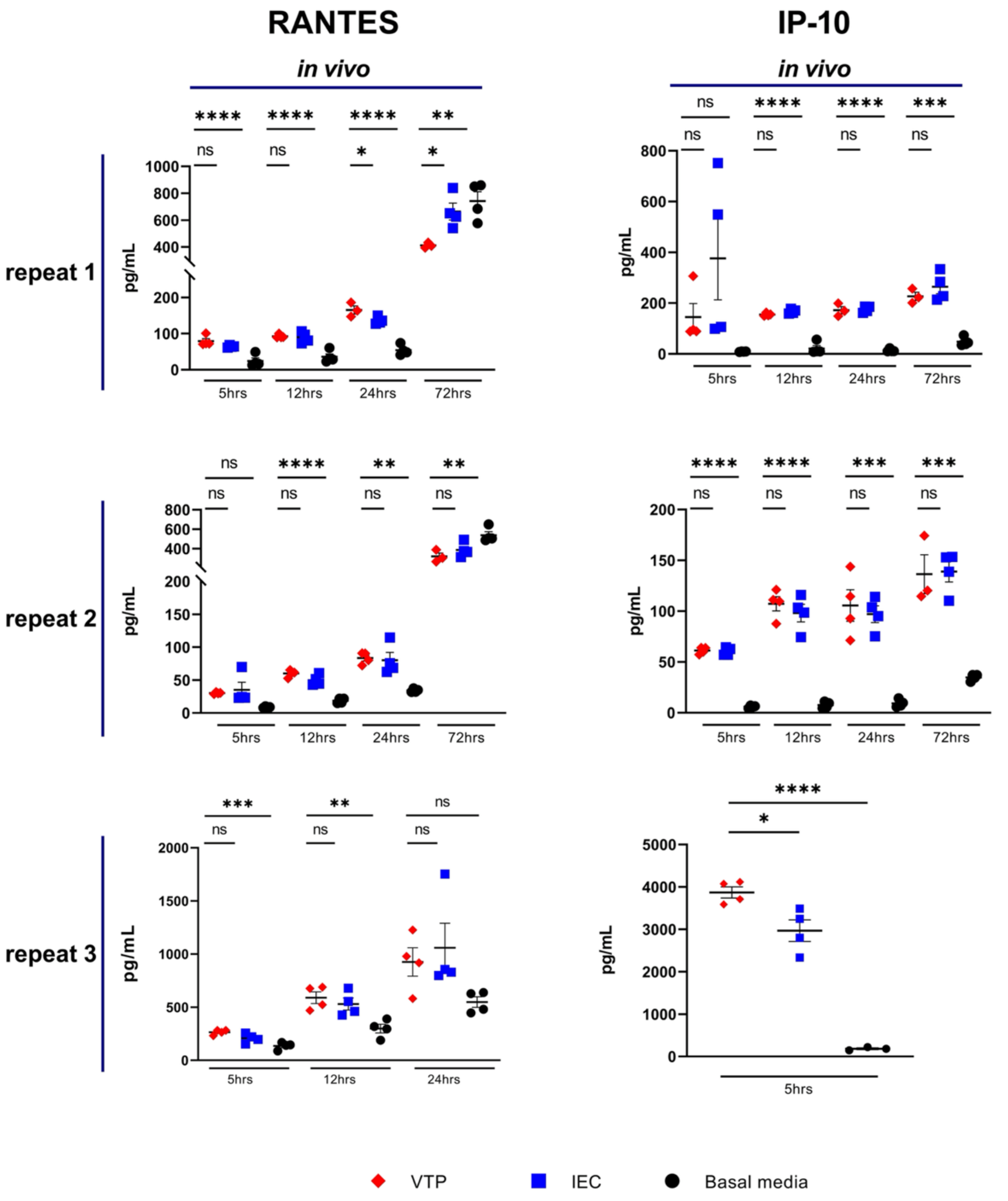


**Supplementary Figure 10.** Concentrations (pg/mL) RANTES (CCL5) and IP-10 secreted by hCMEC/d3 cells exposed to viable *in vivo T. pallidum*. The infection extract control and basal media were included as controls. Culture supernatant was sampled at various timepoints, and secreted cytokines were quantified using a multiplexed cytometric bead array. The upper limit of quantitation (ULOQ) was 5000pg/mL; however, for repeat 3, all IP-10 VTP and IEC samples after 5 hours, and all RANTES samples at 72 hours were above the ULOQ and were therefore excluded from the analysis. Statistical analysis was completed using one-way ANOVA followed by Dunnetts multiple comparison. Significant differences are represented as follows: * = p ≤0.05, ** = p ≤0.01, *** = p ≤0.001, **** = p <0.0001, ns = not significant.

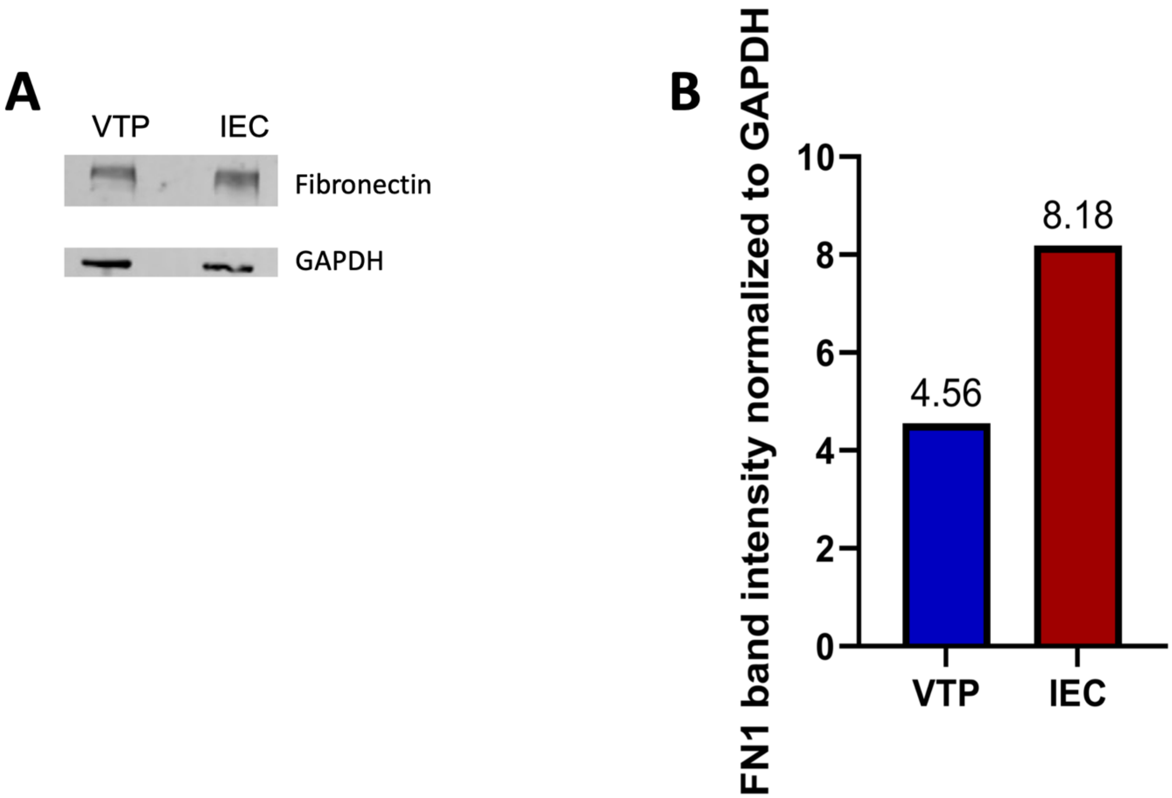


**Supplementary Figure 11.** (**A**) Western blot of human fibronectin (FN1) from hCMEC/d3 endothelial cells exposed to viable *T. pallidum* (VTP) at a MOI of 30, or infection extract control (IEC). Three independent biological replicates of each condition (VTP or IEC) were pooled in equal concentration, and 13.5 µg total protein was added to each lane. (**B**) Band intensity of fibronectin normalized to GAPDH band intensity via Licor Image studio version 5.2.

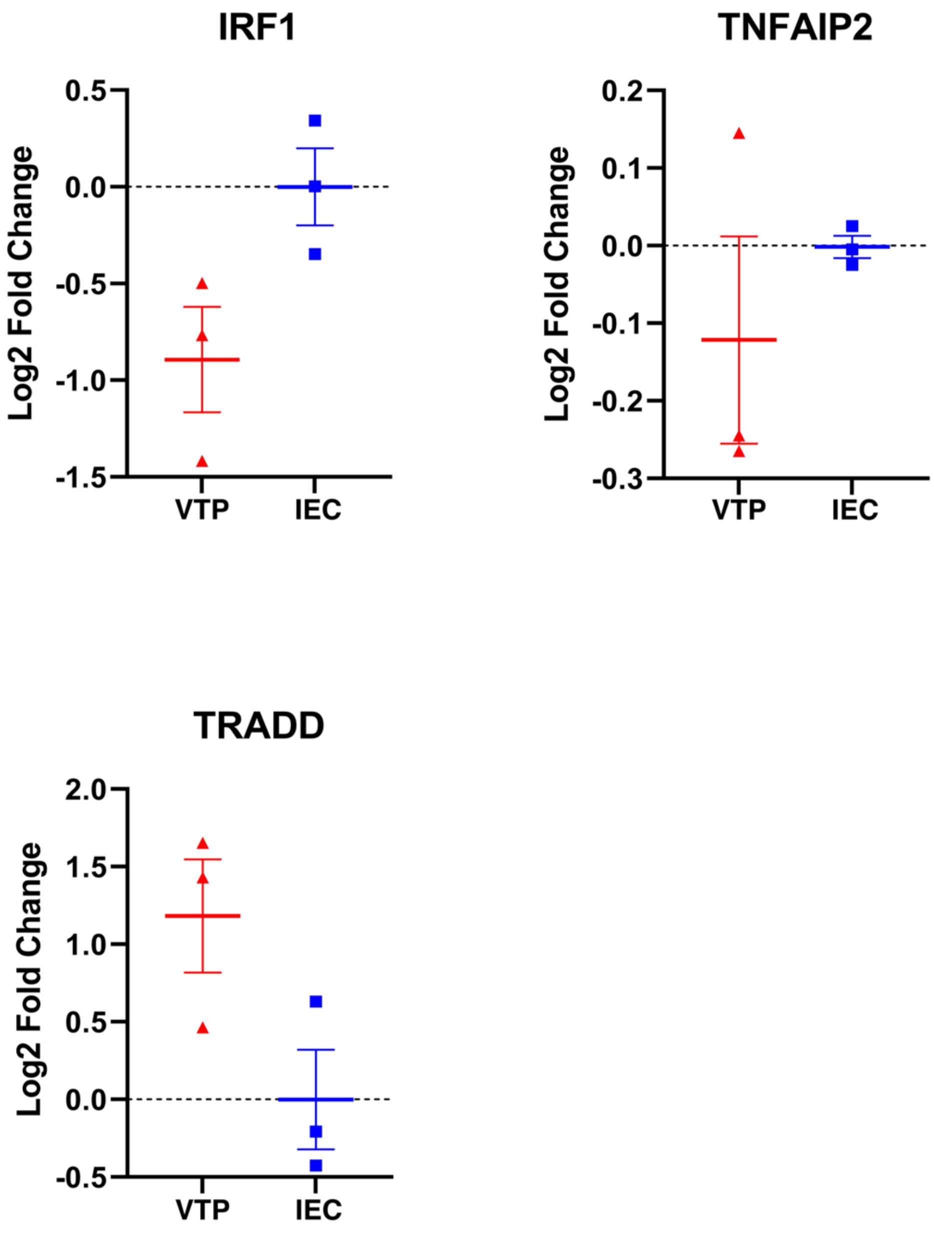


**Supplementary Figure 12.** RT-qPCR analysis of target genes from BEC exposed to *T. pallidum* or our infection extract control. Each datapoint represents an in individual biological replicate. Data were analyzed by the $2^{-\Delta\Delta Cq}$ method normalized to GAPDH mRNA.
